# Snail synchronizes endocycling in a TOR-dependent manner to coordinate entry and escape from endoreplication pausing during the *Drosophila* critical weight checkpoint

**DOI:** 10.1371/journal.pbio.3000609

**Published:** 2020-02-25

**Authors:** Jie Zeng, Nhan Huynh, Brian Phelps, Kirst King-Jones

**Affiliations:** Department of Biological Sciences, University of Alberta, Edmonton, Canada; University of Utah, UNITED STATES

## Abstract

The final body size of any given individual underlies both genetic and environmental constraints. Both mammals and insects use target of rapamycin (TOR) and insulin signaling pathways to coordinate growth with nutrition. In holometabolous insects, the growth period is terminated through a cascade of peptide and steroid hormones that end larval feeding behavior and trigger metamorphosis, a nonfeeding stage during which the larval body plan is remodeled to produce an adult. This irreversible decision, termed the critical weight (CW) checkpoint, ensures that larvae have acquired sufficient nutrients to complete and survive development to adulthood. How insects assess body size via the CW checkpoint is still poorly understood on the molecular level. We show here that the *Drosophila* transcription factor Snail plays a key role in this process. Before and during the CW checkpoint, *snail* is highly expressed in the larval prothoracic gland (PG), an endocrine tissue undergoing endoreplication and primarily dedicated to the production of the steroid hormone ecdysone. We observed two Snail peaks in the PG, one before and one after the molt from the second to the third instar. Remarkably, these Snail peaks coincide with two peaks of PG cells entering S phase and a slowing of DNA synthesis between the peaks. Interestingly, the second Snail peak occurs at the exit of the CW checkpoint. Snail levels then decline continuously, and endoreplication becomes nonsynchronized in the PG after the CW checkpoint. This suggests that the synchronization of PG cells into S phase via Snail represents the mechanistic link used to terminate the CW checkpoint. Indeed, PG-specific loss of *snail* function prior to the CW checkpoint causes larval arrest due to a cessation of endoreplication in PG cells, whereas impairing *snail* after the CW checkpoint no longer affected endoreplication and further development. During the CW window, starvation or loss of TOR signaling disrupted the formation of Snail peaks and endocycle synchronization, whereas later starvation had no effect on *snail* expression. Taken together, our data demonstrate that insects use the TOR pathway to assess nutrient status during larval development to regulate Snail in ecdysone-producing cells as an effector protein to coordinate endoreplication and CW attainment.

## Abstract 2

During *Drosophila* development, the time window when larvae assess their readiness for metamorphosis is marked by slowing of cell growth in the prothoracic gland that produces the molting hormone; cell growth (via DNA endoreplication) then increases, allowing the production of the amount of hormone required to trigger metamorphosis. This study shows that these processes depend on the transcription factor Snail.

## Introduction

Steroid hormones are phylogenetically ancient signaling molecules found in animals and plants alike, where they control a wide range of developmental and physiological processes, such as sexual maturation, reproduction, glucose, and cholesterol metabolism as well as inflammatory responses [1,2]. In *Drosophila melanogaster*, the major steroid hormones are known as ecdysteroids, hereafter referred to as ecdysone, including the prohormone α-ecdysone and the biologically active form 20-hydroxyecdysone (20E). Pulses of ecdysone trigger all developmental transitions, including the molts and pupation in insects [3]. Like vertebrate steroids, ecdysone is synthesized from cholesterol or other suitable sterols in specialized endocrine tissues via several enzymatic steps. In *Drosophila* larvae, the ecdysone-producing tissue is the prothoracic gland (PG), which is part of a larger tissue called the ring gland (RG), the principal neuroendocrine organ in *Drosophila* larvae. Ecdysone biosynthetic enzymes are largely PG-specific, and some are well-characterized [4]. Neverland (Nvd), for instance, carries out the first step and converts cholesterol to 7-dehydrocholesterol (7DC) [5], whereas the last three steps that result in α-ecdysone require Phantom (Phm), Disembodied (Dib), and Shadow (Sad) [6–8]. The intermediate steps that metabolize 7DC to 5ß-ketodiol are poorly understood and require at least three enzymes, Shroud (Sro), Spookier (Spok), and Cyp6t3 [9–11]. Both internal (e.g., the integrity of the larval tissues) and external cues (e.g., nutrient status) are transduced through a series of cellular signaling pathways in the PG and ensure that the larva is ready to produce an ecdysone pulse [12,13]. The known regulatory pathways that act on the PG to control ecdysone biosynthesis include prothoracicotropic hormone (PTTH)/MAPK signaling, transforming growth factor beta (TGF-β)/Activin signaling, nitric oxide (NO), the circadian machinery, insulin/IGF signaling (IIS), and target of rapamycin (TOR) signaling [14–18].

Unlike vertebrates, growth and maturation are separated in holometabolous insects, in which growth occurs during larval stages, whereas maturation takes place during metamorphosis [19,20]. In holometabolous insects, larvae are committed to metamorphosis (maturation) once critical weight (CW) is attained [21]. CW was first described by Nijhout and Williams in *Manduca sexta* [22]. This study found that when larvae were starved before CW is attained, further development was delayed until nutrients become available again. However, once CW was attained, larvae committed irreversibly to metamorphosis and pupariated on schedule, regardless of subsequent nutritional conditions, and thus acquired the competence to produce a major pulse of ecdysone at the end of larval life to trigger metamorphosis [12,19,23–25]. In *Drosophila*, various studies have demonstrated that organ growth and patterning (e.g., eyes or wing imaginal discs) respond differently to nutrient status depending on whether CW has been attained and that these distinct responses are ecdysone-regulated [26–29]. Taken together, the CW is a true physiological checkpoint that allows the study of the molecular mechanisms by which animals assess their nutrient status and body size.

Larvae typically attain CW in the early stage of the last larval instar [20], which is approximately 8–10 hr after the second instar (L2) to third instar (L3) molt in *Drosophila*. But the exact timing of CW attainment depends on diverse factors including temperature [30], oxygen availability [31], sex [32], and imaginal disc integrity [33]. Moreover, in *Drosophila*, the CW checkpoint overlaps with another checkpoint termed minimum viable weight, which is defined as the body weight at which larvae survive starvation and can pupate [34,35]. As such, minimum viable weight is often easier to measure than CW [19,24] and is typically used interchangeably with CW in *Drosophila*. In this manuscript, we will be referring to both definitions simply as “CW.”

Although several signaling pathways—most prominently TOR and IIS signaling—affect the CW checkpoint [18,36–39], the exact molecular mechanisms by which CW is determined remain largely unclear. A minor ecdysone pulse at about 8 hr after the L2/L3 molt is linked to the CW checkpoint [25], since starving larvae prior to CW abrogated this pulse, whereas feeding larvae with ecdysone rescued the developmental delay caused by pre-CW starvation [40]. These data suggest that ecdysone may induce CW attainment; however, it has never been firmly established that CW attainment is the result of an ecdysone pulse at this time [40]. The size of the PG has been suggested to determine CW [37], but not all instances that affect PG size necessarily perturb the CW checkpoint [38]. A recent study showed that endoreplication (a cell cycle variant that consists of only alternating S and G phase without cell division) of PG cells may be an important part of the molecular basis of CW checkpoint [23]. The study found that the DNA content value (C-value) of PG cells from post-CW larvae invariably exceeded 16, suggesting that the PG endoreplication status was tightly coupled to fulfilling the CW checkpoint. Also, the authors showed that endoreplication was dependent on nutrient status, since starvation or PG-specific loss of the nutrient sensor, TOR, arrested the PG endocycle at 16C [23]. This is consistent with earlier findings that TOR signaling is essential for ecdysone production and metamorphosis [18]. However, the dependency of endoreplication on TOR was not seen after the attainment of CW [18,23]. These findings provide a framework for understanding the mechanisms underlying the CW checkpoint that involves (1) a downstream component that irreversibly up-regulates ecdysone production after CW attainment to trigger metamorphosis; (2) a temporary window that allows to couple nutrient sensing in the larval PG to ecdysone synthesis; and (3) the potential importance of endoreplication as a signal that is linked to the CW checkpoint.

The *snail* gene family encodes zinc finger transcription factors that are conserved across metazoans [41,42]. The first described member, *snail*, was identified in *Drosophila* in the Nobel prize–winning Heidelberg screen [43]. *Drosophila* Snail is involved in establishing the mesoderm-neuroectoderm boundary [44–46] and promoting ventral cell invagination [47] during embryogenesis. Snail is also required for the formation of the RG during embryonic development, as well as for specifying RG cell identity [48]. We report here that Snail is part of the molecular mechanism that underlies the CW checkpoint. Our initial observation was that *snail* was highly expressed in early L3 RGs (4–8 hr after the molt to L3) but strongly down-regulated in late L3 RGs, suggesting a role for Snail during the CW checkpoint rather than being required for later ecdysone pulses [49]. In addition, both PG-specific *snail* overexpression or loss of *snail* function interfered with ecdysone biosynthesis and metamorphosis. Remarkably, immunostaining revealed that Snail was detectable only in a subset of PG nuclei at most time points, which was strikingly similar to the pattern of S-phase cells in the PG. We identified two Snail peaks, one at 17–18 hr after the molt to the L2 stage (4–5 hr prior to the L2/L3 molt) and one around 8–12 hr after the molt to L3. These peaks coincided with two waves of endocycle progression in the PG, suggesting that Snail peaks are important for endoreplication. Also, the second peak coincided with the attainment of CW. In line with these concurrences, PG-specific loss of *snail* function caused endoreplication arrest right before CW attainment, which stalled further larval development. We also showed that the nuclear presence of Snail protein in the PG is responsive to the nutrient sensor TOR, as well as starvation. Therefore, we conclude that Snail acts as a regulator of nutrient-dependent endoreplication of PG cells. Lastly, the presence of Snail in PG nuclei (this study) and mRNA levels of *snail* in RG samples [49] decline rapidly after the CW checkpoint, consistent with our findings that *snail* is no longer essential for endoreplication and metamorphosis after the CW checkpoint is fulfilled. Taken together, *Drosophila* Snail represents a new component of the CW checkpoint that links nutrient control and endoreplication to the CW checkpoint.

## Results

### *snail* is expressed in the larval PG

**Fig 1:**
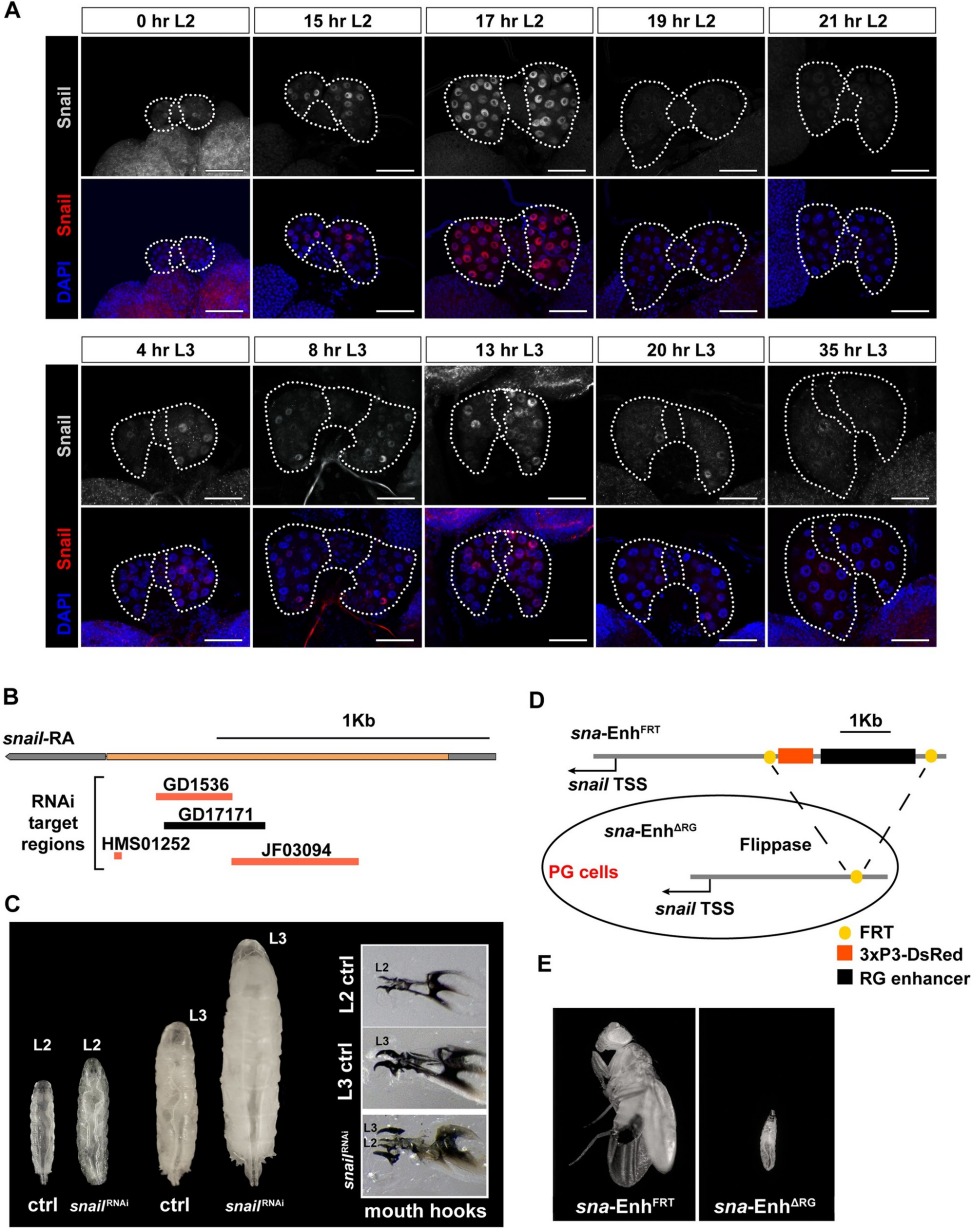
*snail* is dynamically expressed in the larval PG and required in the PG during development. **(A)** Single-plane confocal images showing RGs stained with anti-Snail antibodies and DAPI. *w*^*1118*^ larvae were dissected and examined at various time points during larval stages. The PG and CA are outlined by a white dotted line. Eight to 10 RGs were examined for each condition. Scale bar: 50 μm. **(B)** The *Drosophila snail* transcription unit and target sequences of existing *snail*-RNAi lines. Noncoding sequences are in gray, and coding region is in orange. GD1536, GD17171 (from VDRC), HMS01252, and JF03094 (from TRiP) are transgenes that produce dsRNA. **(C)** PG-specific knockdown of *snail* caused developmental arrest in L2 and L3 larvae. Control (“ctrl”) L2 were collected at 64 hr AED, whereas *snail*-RNAi L2 larvae that failed to molt to the L3 stage were collected at 86 hr AED when controls had molted to L3. Control L3 larvae were removed at 92 hr AED, and the arrested *snail*-RNAi L3 were collected 4 d later when controls had pupariated. Control: *UAS*-*Dicer2*; *phm22*-*Gal4*>*w*^*1118*^. *snail*^RNAi^: *UAS*-*Dicer2*; *phm22*-*Gal4*>*UAS*-*snail-*RNAi (VDRC#50003). **(D)** Schematic of the *sna*-Enh^FRT^ allele we used to generate mosaics in which the RG enhancer of *snail* was deleted (*sna*-Enh^ΔRG^) because of PG-specific expression of Flippase. **(E)** Survival of *sna*-Enh^FRT^ animals and *sna*-Enh^ΔRG^ animals. *sna*-Enh^FRT^ animals can develop normally to adulthood, whereas *sna*-Enh^ΔRG^ animals arrested development as early L2. AED, after egg deposition; CA, corpus allatum; dsRNA, double-strand RNA; FRT, flippase recognition target; L2, second instar; L3, third instar; PG, prothoracic gland; RG, ring gland; RNAi, RNA interference; TRiP, Transgenic RNAi Resource Project; TSS, transcription start site; VDRC, Vienna *Drosophila* Resource Center.

In a previous study from our lab, we showed that *snail* expression was >20-fold higher in RG samples than in whole-body samples, at least in early L3 larvae [49]. *snail* expression then rapidly declined in mid and late L3. Since the RG comprises three endocrine glands, we first examined whether *snail* is expressed in the PG, for which we used anti-Snail antibodies to conduct immunofluorescent detection for a series of larval time points. We found that Snail protein was only detectable in PG nuclei and appeared to be absent in the two adjacent glands, the corpus allatum (CA) and the corpus cardiacum (CC) (Fig 1A). Surprisingly, Snail exhibited a mosaic pattern in the PG, which we will discuss in a later section. Another notable observation was that Snail levels showed a strict temporal pattern, as we detected two distinct peaks, one of which occurred in L2 (17–18 hr after the L1/L2 molt) and the other in L3 (8–12 hr after the L2/L3 molt). However, the two peaks were distinct, since the L2 peak showed presence of Snail in nearly all nuclei, whereas the L3 peak was typically limited to a subset of nuclei. Moreover, consistent with our earlier finding that *snail* mRNA levels decline in the RG during the L3 stage [49], Snail protein levels were substantially reduced in PGs from late L3 larvae (after 36 hr L3) (Fig 1A). We then confirmed these observations with a transgenic fly line expressing a GFP-tagged *snail* genomic P[acman] construct (harboring most or all of the *snail* regulatory regions) via GFP antibody staining (S1 Fig) [50]. The presence of one Snail peak prior to the last larval instar and one during the CW checkpoint raised the idea that Snail has key roles in regulating CW. Although it appears plausible that the second peak controls the exit from this checkpoint, the role of the first Snail peak remains unclear.

### PG-specific disruption of *snail* function caused developmental arrest due to failure in ecdysone production

To examine the importance of *snail* function in the larval PG, we disrupted *snail* via PG-specific RNA interference (RNAi; Vienna *Drosophila* Resource Center [VDRC] #50003, with *phm22*-Gal4, hereafter *PG>*). A quantitative PCR (qPCR) analysis showed that *snail*-RNAi line was functional, since *snail* transcripts levels were approximately 70% reduced in RG samples isolated from PG>*snail*-RNAi larvae compared to those of controls (S2A Fig). Immunolabeling also failed to detect Snail in *snail*-RNAi PGs (S2B Fig). PG-specific *snail*-RNAi caused larval arrest in which about 20% of the population stalled development as L2, and the remaining 80% reached, but did not progress beyond, the L3 stage. This bimodal lethal phase was consistent with the two expression peaks we described previously. A closer examination of the arrested L2 larvae revealed that they had molting defects. Specifically, L2 larvae showed both an L2 mouth hook and a larger L3 mouth hook (referred to as the “double mouth hook” phenotype); however, these larvae failed to complete the molt to L3 (Fig 1C). The arrested L3 larvae never engaged in wandering behavior and instead continued feeding, which resulted in excess growth with a concomitant giant L3 phenotype (Fig 1C). Both growth and mouth hook phenotypes are typically caused by a lack or reduction of ecdysone production during larval development [9,51–54], suggesting that *snail* function was required for proper ecdysone biosynthesis. To validate the RNAi results, we generated a PG-specific *snail*-deletion line using CRISPR/Cas9. For this, we flanked the *snail* RG enhancer with flippase (FLP) recognition target (FRT) sites, allowing us to delete the sequence via PG-specific FLP expression. Removal of the *snail* RG enhancer (*sna*-Enh^ΔRG^) appeared to be a bit stronger compared to the RNAi line, as it caused lethality during the L2 stage (Fig 1E).

**Fig 2:**
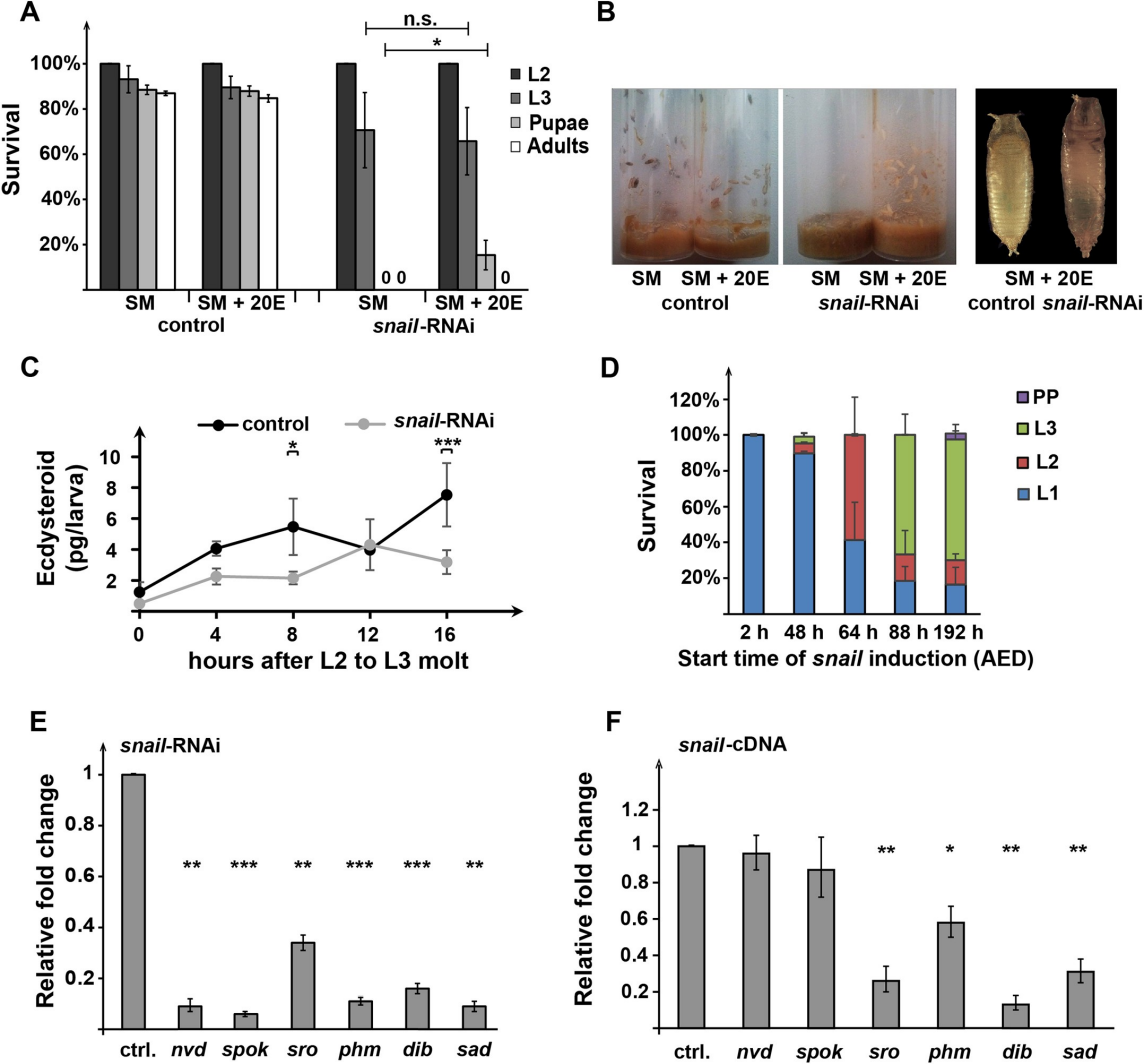
Ecdysone production is dependent on proper *snail* levels in the PG. **(A)** Developmental arrest caused by PG>*snail*-RNAi was partially rescued by ecdysone feeding. Bar graphs show the percentage of larvae that survived to the indicated stages. Fifty L2 larvae of each genotype were used as a starting population. Error bars represent standard deviation. *p < 0.05 (one-way ANOVA). **(B)** Left panel: example vials from experiments shown in (A). Larvae on the vial wall indicate that wandering behavior had occurred. Right panel: control and *snail*-RNAi PP reared on 20E supplemented food. **(C)** Ecdysteroid titers in whole-body larvae were measured at various time points during early L3 stage. Error bars represent standard deviation. *p < 0.5, ***p < 0.001 (two-way ANOVA). **(D)** Survival rates of animals conditionally expressing *snail* in the PG. *Gal80*^*ts*^; *phm22*>*snail*-cDNA embryos/larvae were initially reared at 18°C and then shifted to the restrictive temperate (29°C) at the indicated time points to induce the expression of the *snail*-cDNA. Bar graphs show the percentage of animals that reach indicated developmental stages. Error bars represent standard deviation. **(E)** qPCR analysis showing the expression levels of six ecdysone biosynthetic genes in *snail*-RNAi RGs. RGs were dissected at 24 hr after L2 to L3 molt. **(F)** qPCR results showing the expression of the six ecdysone biosynthetic genes in hs>*snail*-cDNA RGs. The expression of each gene in *snail* overexpression was normalized to the expression in the control (*hs*-*Gal4*> *y*^*[1]*^w^*[67c23]*^). (A-C, E) Control/ctrl.: UAS-*Dicer2*; *phm22-Gal4*>*w*^*1118*^. *snail*-RNAi: UAS-*Dicer2*; *phm22-Gal4*>UAS-*snail*-RNAi. (E and F) Error bar represents the 95% confidence interval. *p < 0.05, **p < 0.01, ***p < 0.001 (Student *t* test). Underlying data for this figure can be found in S1 Data. 20E, 20-hydroxyecdysone; AED, after egg deposition; *dib*, *disembodied*; *Gal80*^*ts*^, Gal80 temperature-sensitive allele; L1, first instar; L2, second instar; L3, third instar; *nvd*, *neverland*; n.s., not significant; PP, pupae; PG, prothoracic gland; *phm*, *phantom*; PP, pupae; qPCR, quantitative PCR; RG, ring gland; RNAi, RNA interference; *sad*, *shadow*; SM, agar-cornmeal-based standard medium; *spok*, *spookier*; *sro*, *shroud*.

If the developmental arrest in PG>*snail*-RNAi animals was—at least in part—caused by a reduction in ecdysone production, we expected to see a rescue when the biologically active form of ecdysone, 20E, was added to the fly medium. Indeed, we observed that about 50% of the *snail*-RNAi L3 started wandering behavior in the presence of 20E, some of which formed pupae, which accounted for 15.4% (±6.5%) of the starting population (Fig 2A). However, these pupae were not healthy enough to develop into adults (Fig 2A and 2B). We did not observe any improvement of L2 lethality in the *snail*-RNAi animals by 20E feeding (Fig 2A). We concluded that 20E partially rescued the PG*>snail*-RNAi phenotype, suggesting *snail* is—directly or indirectly—required for ecdysteroidogenesis.

To determine the extent by which *snail*-RNAi affected ecdysone production, we measured ecdysone titers in whole-body larval samples collected during early L3 stages (at 0, 4, 8, 12, and 16 hr L3). We chose these time points because the majority (80%) of the *snail*-RNAi larvae reached the L3 stage but never engaged in wandering behavior, suggesting that *snail* has critical functions in early L3 prior to wandering behavior. In addition, these five time points capture the second Snail peak as well as the minor ecdysone pulse (at 8 hr L3) during the CW checkpoint [25]. We found that *snail*-RNAi larvae produced significantly less ecdysone during these times, with the minor ecdysone peak (at 8 hr L3) being submaximal and delayed by 4 hr (Fig 2C). At 16 hr L3, ecdysone concentrations rose again in controls, consistent with the beginning of the second minor peak [25]; however, this failed to occur in *snail*-RNAi larvae. Taken together, overall ecdysone levels were significantly reduced in PG>*snail*-RNAi larvae during the CW window.

We reasoned that ectopic expression of *snail* in the PG may also reveal informative phenotypes, since transcription factor overexpression often perturbs developmental processes in *Drosophila* [9,55,56]. We showed that overexpressing *snail* using the *phm22*-Gal4 driver resulted in 100% first instar (L1) larval arrest (author’s observation), possibly caused by a lack of ecdysone required for the molt from L1 to L2. Since PG-*snail* overexpression caused lethality very early on during development, we induced *snail* overexpression conditionally in the PG at various larval stages using the Gal80 temperature-sensitive allele (Gal80^ts^) [57]. Gal80^ts^ protein prevented *snail*-cDNA expression in the PG at 18°C, whereas the *snail*-cDNA was expressed when the temperature was shifted to 29°C. We found that when *phm22*,*Gal80*^*ts*^>*snail*-cDNA animals were shifted to 29°C at 48 hr after egg deposition (AED) [58], 90% of the animals were arrested at L1 (Fig 2D). At 18°C, development was slowed, and most animals were in early L1 at 48 hr AED. This suggested that *snail* overexpression prevented ecdysone production in mid-L1. Moreover, inducing *snail* expression later at 88 hr AED (when animals are early L2) caused mostly L3 arrest (Fig 2D), which suggested that when *snail* overexpression was in effect after an ecdysone pulse had already formed, larvae would be arrested in the next instar (Fig 2D). These observations suggested that increased *snail* levels directly repress ecdysone production, consistent with our observation that *snail* is dramatically down-regulated prior to the major ecdysone pulse in late L3 (Fig 1A) and, as such, may be a prerequisite to allow for the drastic up-regulation of ecdysone production required for triggerring metamorphosis. Taken together, results of knockdown and overexpression of *snail* demonstrated that this transcription factor must be tightly controlled to allow for proper ecdysone production and metamorphosis.

### Dynamic expression of *snail* needs to be maintained in the PG for proper expression of the biosynthetic genes

Given the importance of *snail* function for ecdysone production, we wondered whether the expression of the ecdysone biosynthetic genes was dependent on Snail in the PG. Therefore, we performed RNA sequencing (RNA-Seq) analyses with hand-dissected RG samples from PG>*snail*-RNAi and control larvae as well as heat shock–induced (hs) *snail*-overexpression animals. All samples were collected from L3 larvae at 24 hr after the molt, because RGs from these larvae are large enough for dissection, and larvae undergo minimal physiological changes compared to later time points closer to puparium formation when a major ecdysone pulse occurs. When we performed gene ontology (GO) term enrichment analysis via DAVID and STRING [59,60], of the 201 >2-fold down-regulated genes in *snail*-RNAi, the only significant GO term was "Ecdysteroid biosynthetic process" (Table 1), suggesting that ecdysone biosynthetic genes were selectivly down-regulated in the absence of functional *snail*. We confirmed this coordinated down-regulation via qPCR, which showed strongly reduced levels for *spok*, *nvd*, *phm*, *sro*, *sad*, and *dib* in *snail*-RNAi RG samples (Fig 2E; S1 Table).

**Table 1:** GO terms enrichment analysis of the deregulated genes in PG>*snail-*RNAi as well as *hs*>*snail*-cDNA RGs.

#Pathway ID	Pathway description	FDR	Gene names
>2 fold down-regulated genes in PG>*snail*-RNAi samples			
GO_biological process	Ecdysteroid biosynthetic process	9.6E-07	*GstE14*, *npc1a*, *npc2b*, *dib*, *nvd*, *phm*, *sad*, *spok*
>2 fold up-regulated genes in PG>*snail*-RNAi samples			
GO_molecular function	Alkaline phosphatase activity/Folate biosynthesis	5.5E-03	*Alp4*, *CG10592*, *CG3264*, *CG3292*, *CG5150*, *CG5361*
KEGG	Metabolism of xenobiotics by cytochrome P450/ Glutathione metabolism	1.6E-03	*CG4302*, *CG5999*, *GstD2*, *GstD5*, *GstD7*, *GstE1*, *GstE10*, *GstE9*, *Ugt35b*
InterPro domain	Protein of unknown function DUF227/CHK kinase-like	1.0E-02	*CG10513*, *CG10514*, *CG10559*, *CG11892*, *CG11893*, *CG32195*, *CG33510*, *CG6834*, *CG9259*
>2 fold down-regulated genes in hs>*snail*-cDNA samples			
Interpro domain	LPS-induced tumor necrosis factor alpha factor	1.2E-02	*CG13510*, *CG13559*, *CG30269*, *CG30273*, *CG32280*, *CG42566*, *CG43326*
>2 fold up-regulated genes in hs>*snail*-cDNA samples			
GO_biological process	Response to bacterium	6.5E-03	*CecA1*, *IM14*, *IM23*, *IM4*, *Mtk*, *Sid*, *Yp3*
KEGG	Neuroactive ligand-receptor interaction	3.2E-03	*AdoR*, *AstA-R2*, *CG30031*, *NPFR*, *deltaTry*, *gammaTry*

GO enrichment analysis was carried out using DAVID and STRING.

Abbreviations: FDR, false discovery rate; GO, gene ontology; hs, heat shock–induced; LPS, lipopolysaccharide; PG, prothoracic gland; RG, ring gland; RNAi, RNA interference.

For *snail* overexpression, a single heat shock (at 37°C for 50 min) was carried out before 24 hr L3, after which larvae were allowed to recover at 25°C for 6 hr and dissected at 24 hr L3. Given the short period of *snail* overexpression, we expected that secondary effects caused by the overexpression would be limited, and differentially expressed genes identified by RNA-Seq would be primarily caused directly by Snail. When we compared expression profiles to controls, we noticed that *dib* was ranked the 10th-most down-regulated gene (approximately 17-fold down-regulated, S3 Table) among 245 genes with >3-fold repression. However, ecdysone biosynthetic genes were not enriched as a GO term in this cohort (Table 1). The down-regulation of *dib* was also confirmed via qPCR (Fig 2F). In addition, the expression of *sro* was also significantly affected (about 3-fold) by *snail* overexpression, shown both by RNA-Seq and qPCR (Fig 2F; S4 Table). Taken together, we established that *snail* function is required in the larval PG for ecdysone biosynthesis. In addition, ectopic expression of *snail* strongly inhibited *dib*, an ecdysone biosynthetic gene, suggesting that *snail* may control ecdysone synthesis by regulating at least some Halloween genes in a direct manner.

### *snail* function is required for endocycle progression in PG cells

**Fig 3:**
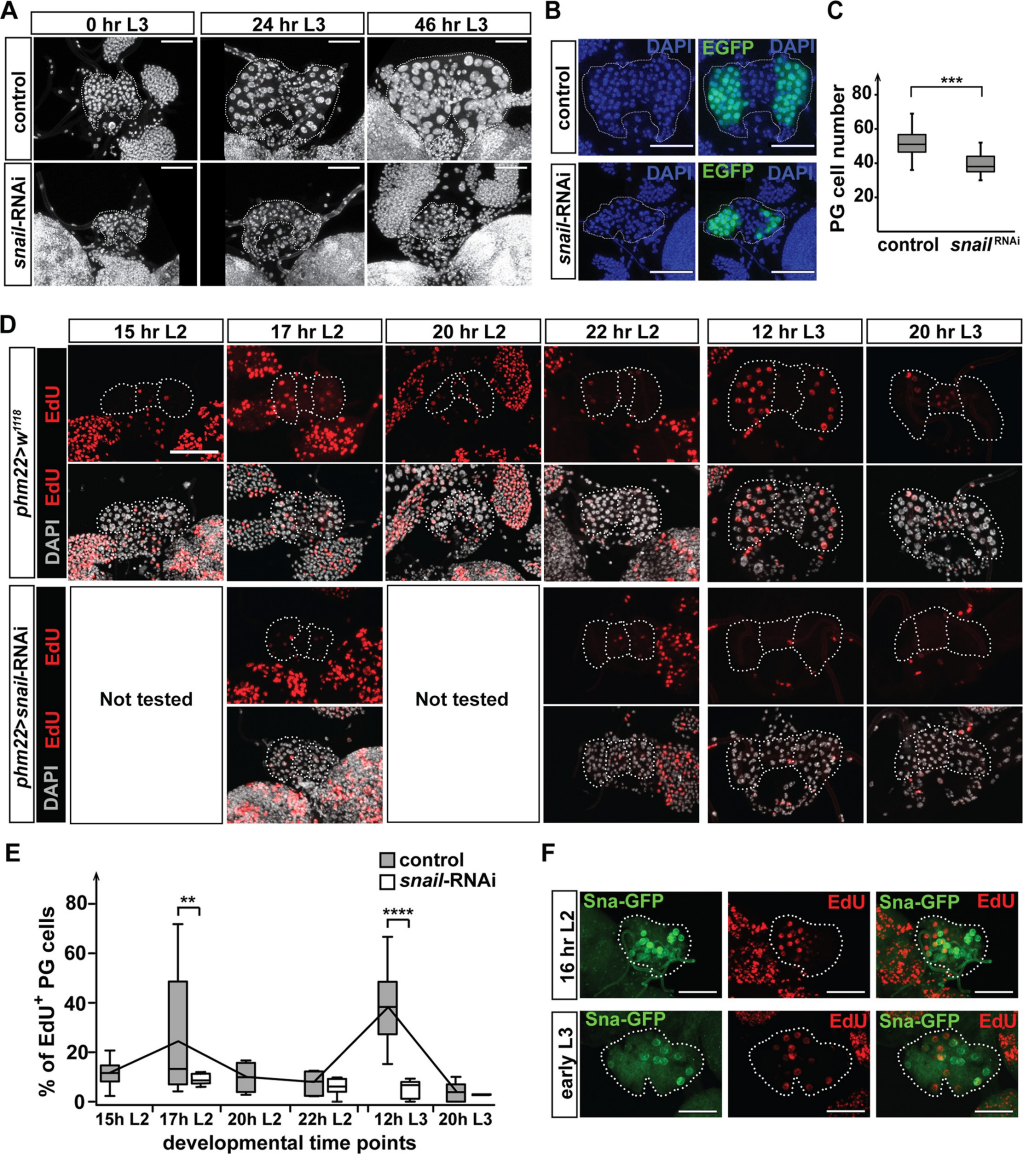
*snail* is required for endocycle progression in the PG. **(A)** Maximal projection of Z-stack confocal images of RGs dissected at various times relative to the L2/L3 molt. Tissues were stained with DAPI to show nuclei. RGs are outlined by white dotted lines. *snail*-RNAi: *UAS*-*Dicer2*; *phm22*-*Gal4*>*UAS*-*snail-*RNAi and control: *UAS*-*Dicer2*; *phm22*-*Gal4*>*w*^*1118*^. **(B)** Maximal projection of the Z-stack confocal images of RGs dissected at the L2/L3 molt. *UAS*-*EGFP* expression was driven by *phm22*-*Gal4* to label PG cells. **(C)** Numbers of PG cells (EGFP+) are shown in the whisker box plot. ***p < 0.001 (based on Student *t* test). (B and C) *snail*-RNAi/*snail*^RNAi^: *UAS*-*Dicer2*; *phm22*-*Gal4*>*UAS*-*snail-*RNAi; *UAS*-*EGFP*. Control: *UAS*-*Dicer2*; *phm22*-*Gal4*>*UAS*-*EGFP*. **(D)** *snail*-RNAi PGs showed a reduction in EdU incorporation at the indicated developmental stages. The PG and CA are outlined by a white dotted line. The percentages of EdU-positive PG cells are shown in box plots **(E)**. The average values of controls at each time point are connected by the black line. **p < 0.01, ****p < 0.0001 (based on multiple *t* test corrected for multiple comparisons using the Holm-Sidak method). **(F)** Maximal projection of Z-stack confocal images showing the Snail-GFP protein distribution as well as the S-phase cells by EdU incorporation. Early L3: 8–12 hr L3 (around the time of CW attainment). Tissues were dissected from *snail*-*gfp* transgenic line. (A, D, and E) Control: *UAS*-*Dicer2*; *phm22*-*Gal4*>*w*^*1118*^. *snail*-RNAi: *UAS*-*Dicer2*; *phm22*-*Gal4*>*UAS*-*snail-*RNAi. (A, D, and F) Scale bars: 50 μm. Underlying data for this figure can be found in S1 Data. CA, corpus allatum; CW, critical weight; EdU, 5-ethynyl-2′-deoxyuridine; EGFP, enhanced GFP; L2, second instar; L3, third instar; PG, prothoracic gland; RG, ring gland; RNAi, RNA interference.

With the exception of ecdysone biosynthesis, we identified no other *snail*-dependent processes in our RNA-Seq analysis. As such, the underlying mechanism by which *snail* regulated ecdysone production remained elusive. To tackle this question, we explored a second phenotype we observed in *snail*-depleted PGs, namely the reduction in the PG cell and nuclear size. Both *snail*-RNAi as well as FLP-mediated deletion of the *snail* RG enhancer (*sna*-Ehn^ΔRG^) resulted in the appearance of small PG nuclei (Fig 3A; S2C–S2E Fig). However, *snail*-RNAi gave more consistent results, as the somatic CRISPR/Cas9 approach resulted in both normal and small nuclei, probably caused by inconsistent deletion events between individual cells (S2C Fig). Despite this, the PG area, as well as the ratio of PG area to CA area in *sna*-Ehn^ΔRG^, was significantly reduced (S2D and S2E Fig), suggesting that PG cell and nuclear size is specifically affected by the disruption of *snail* function. The *Drosophila* larval PG is an endoreplicating tissue where cells undergo alternating S (DNA synthesis) and G (gap) phases without cell divisions, thus resulting in large nuclei with polytene chromosomes. At the end of larval growth, the average PG cell will reach a C-value of 64 [61]. An increase in DNA content typically correlates with increased nuclear size as well as cell size [62–64]. Therefore, we hypothesized that the small size of *snail*-RNAi PG was caused by endocycle arrest. To test this, we quantified the percentage of S-phase cells in both control and *snail*-RNAi PGs by 5-ethynyl-2′-deoxyuridine (EdU) incorporation at several developmental time points during late L2 as well as L3 stages. For the L2 stage, we tested controls at 17 hr L2 when the first Snail peak occurred in the PG, as well as time points flanking this Snail peak (i.e., 15 hr L2, 20 hr L2, and 22 hr L2) (Fig 3D). A previous study established that a single round of endoreplication in the PG is coupled with the CW checkpoint [23]. Therefore, for the L3 stage, we tested 12 hr L3 (close to the end of the CW checkpoint) and 20 hr L3 (after the entire population has attained CW) (Fig 3D). Our results demonstrated that the endocycle was not synchronized in the PG, which is consistent with previous findings. Specifically, on average there are only 7.6% of the control PG cells in S phase at the end of the L2 stage (22 hr L2), which we interpreted as the stochastic/basal rate of PG cells entering S phase [23]. We observed a rise in the percentage of S-phase cells to about 35.2% at 12 hr L3, and we also noticed a prior wave of endocycle progression around 17 hr L2 with approximately 27% of PG cells in S phase (Fig 3D and 3E). Taken together, this indicates that PG endoreplication occurs in a more synchronized and coordinated manner at these two developmental time points. When we conducted these experiments in PG>*snail*-RNAi PGs, the percentage of EdU-positive cells remained low at all time points (never >12%) and PG cells did not display peaks of endoreplication at 17 hr L2 and 12 hr L3 (Fig 3D and 3E). Moreover, we noticed that the PG cell number in *snail*-RNAi animals was reduced (Fig 3B and 3C). This is consistent with the finding that PG-specific RNAi of *Cyclin-dependent kinase 2*, *Cyclin* E (*CycE*), *double parked* (*dup*), *Proliferating cell nuclear antigen*, or *Cullin* 4 all major regulators of endoreplication, caused not only reduced DNA content in PG cells but also reduced PG cell count [23]. Thus, our results indicated that *snail* is a novel regulator of endoreplication. Strikingly, the mosaic Snail pattern in the PG resembled the EdU labeling pattern, and there was a clear positive correlation between the number of Snail-positive cells around 17 hr L2 and 12 hr L3 and the number of EdU-positive cells (Figs 1A and 3E; S1 Fig). This raised the possibility that Snail levels in a given PG cell are cyclical, consistent with the oscillatory behavior of cell cycle regulators.

We then asked whether Snail is exclusively present during the S phase or G phase during each round of the endocycle. Therefore, we performed double labeling of EdU and Snail using the transgenic line carrying the *snail*-*GFP* genomic clone. Larvae at 16 hr L2 and 8–12 hr L3, which are the two developmental stages with a high proportion of both Snail- and EdU-positive cells in the PG, were dissected, and tissues were allowed for EdU incorporation for 30 min before GFP staining and EdU visualization. In general, we found that *snail*-expressing cells did not overlap with the EdU-positive cells (Fig 3F), strongly suggesting that *snail* is not activated during S phase. Snail is therefore likely induced either right after or before S phase (consistent with the fact that there was occasional overlap between Snail-positive and S-phase cells, Fig 3F).

**Fig 4:**
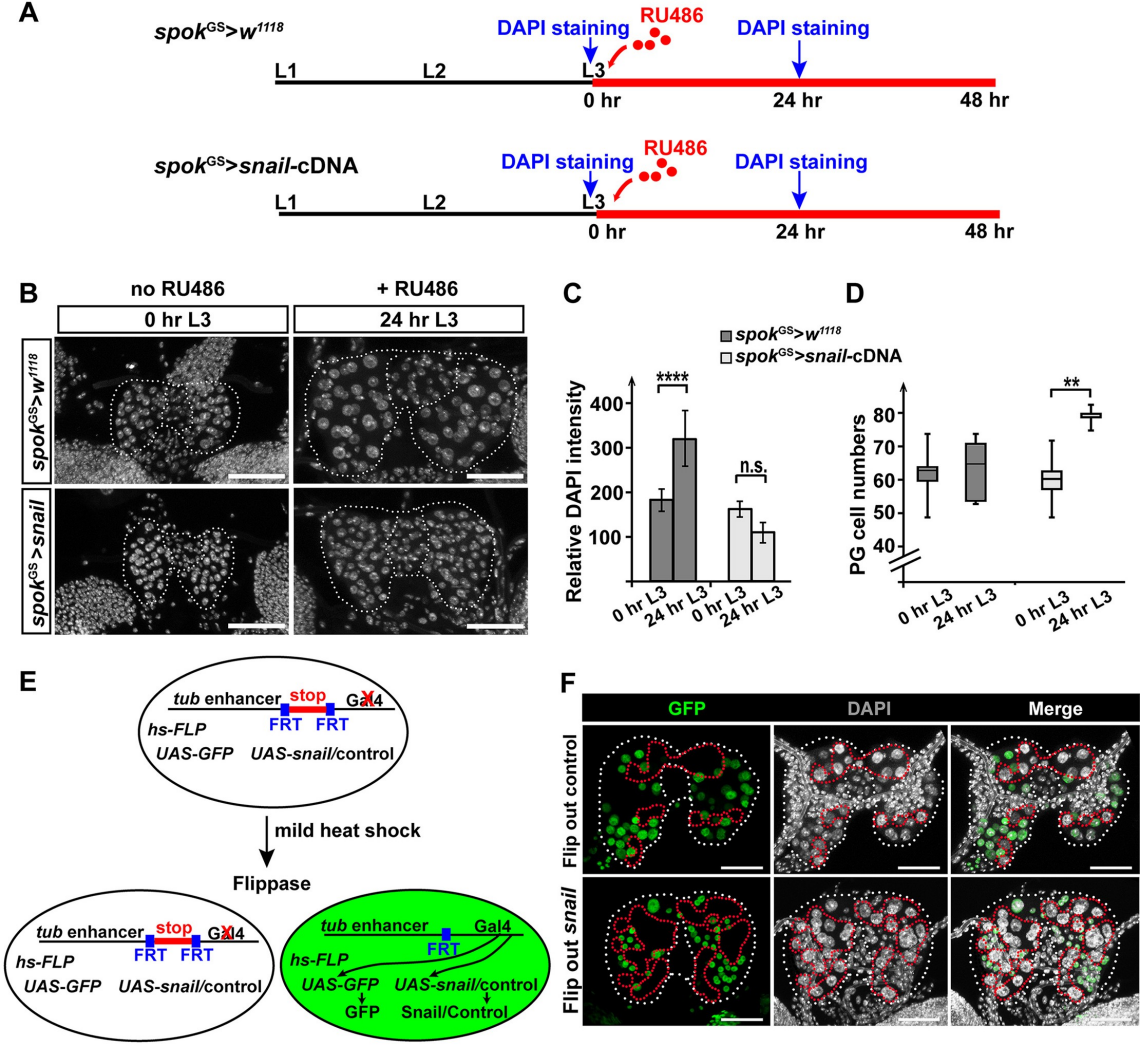
Overexpression of *snail* in the PG inhibits the endocycle progression. **(A)** A schematic illustration showing RU486 feeding procedures for temporal control of *snail* transgene induction. The black line indicates developmental stages prior to RU486 treatment, and the red line indicates when animals were fed with RU486-containing food. Hours labeled with “DAPI staining” indicate time points when RGs were dissected and imaged. **(B)** Maximal projection of Z-stack confocal images showing the size of PG nuclei. Samples were collected and stained with DAPI according to the procedures shown in panel A. Dotted lines mark the boundary between the PG and the CA. **(C)** Relative DNA content per PG nucleus was calculated using the Z-stack images shown in panel B. ****p < 0.0001. **(D)** PG cell numbers were increased in PG>*snail* overexpression animals. PG cell numbers before (0 hr L3) and after (24 hr) RU486 application were quantified for both genotypes. **p < 0.01. **(E)** A schematic illustration of the flip-out-Gal4 system. Blue boxes represent FRT sites. **(F)** Maximal projection of Z-stack confocal images showing the size of the nuclei. Samples were collected and stained with DAPI. Flip out control: *hs-FLP*; *tubulin*-*FRT*-*CD2*-*FRT*-*Gal4*, *UAS*-*GFP*>*y*^*[1]*^ *w*^*[67c23]*^. Flip out *snail*: *hs-FLP*; *tubulin*-*FRT*-*CD2*-*FRT*-*Gal4*, *UAS*-*GFP*>*y*^*[1]*^ *w*^*[67c23]*^*; snail-*cDNA. The white dotted line marks the boundary of the PG and CA. The red dotted line marks the non-GFP cells. (B and F) Scale bar: 50 μm applicable for all the samples shown in the panels. Underlying data for this figure can be found in S1 Data. CA, corpus allatum; FRT, Flippase recognition target; *hs-FLP*, Flippase controlled by a heat shock promoter; L1, first instar; L2, second instar; L3, third instar; n.s., not significant; PG, prothoracic gland; RG, ring gland; stop, a stop codon; *tub*, *tubulin*.

Our results suggested that Snail acts as an endocycle regulator in which oscillatory levels of Snail are linked to cyclical rounds of endoreplication. We hence predicted that overexpression of *snail* in the PG may disrupt the normal cyclical functions of Snail and cause endocycle arrest, similar to *CycE* overexpression [65,66]. To test this, we took advantage of the Gal4-GeneSwitch system to conditionally overexpress *snail* in the PG during the early L3 stage because PG>*snail*-cDNA caused early lethality. The *spok*-GeneSwitch-Gal4 (*spok*^*GS*^*-Gal4*) mediates PG specificity via the *spok* enhancer and temporal control via dietary addition of RU486 [67]. When control animals were fed RU486-containing food at the L2 to L3 molt (0 hr L3) for 24 hr, we detected one round of endoreplication by comparing the DNA contents between 0 hr L3 and 24 hr L3 (Fig 4A–4C). However, when we carried out the same procedure in *spok*^GS^> *snail-*cDNA animals, the DNA content of PG cells remained unchanged during the 24 hr of RU486 application (Fig 4B and 4C), indicating that increasing *snail* levels in this manner blocked endoreplication around the time of CW attainment.

Interestingly, after 24 hr of RU486 administration, the number of PG cells in *spok*^GS^>*snail-*cDNA animals was increased when compared to that of controls (*spok*^GS^> *w*^*1118*^) (Fig 4D). This finding is consistent with the previous report that overexpressing *CycE* (a key driver of endoreplication) in the PG also caused an increase in cell number [23] (S3 Fig). Moreover, the increase in cell number upon *snail* overexpression is also in line with our results that disruption of *snail* function (Fig 3B and 3C) or other endoreplication regulators [23] reduced the number of PG cells.

Since *snail* overexpression in the PG blocks developmental progression (Fig 2D), it was possible that the endocycle arrest in the PG was an unrelated defect caused by delayed development. To rule out this possibility, we took advantage of the flip-out-Gal4 system to overexpress *snail* in a mosaic manner, which allowed us to compare *snail-*overexpressing and control cells within the same tissue. For this, we crossed *hs*-FLP; *tubulin*-FRT-CD2-FRT-Gal4, UAS-GFP flies to UAS-*snail*-cDNA, in which a stop codon was flanked by two FRT sites that normally prevent Gal4 expression [68]. After a mild heat shock in the L1 stage, the stop codon was excised by FLP in a random manner, leading to both *snail*-overexpressing cells (marked by GFP) and cells serving as endogenous controls (non-GFP) (Fig 4E). We dissected the RGs from late-L3 larvae and found that the *snail*-overexpressing cells (GFP+) had smaller nuclei compared to the control (non-GFP) cells (Fig 4F). We also included a control in which cells will express only GFP but not *snail*-cDNA after heat shock. In this scenario, the GFP+ cells had slightly smaller nuclei compared to those of non-GFP cells, suggesting that overexpressing GFP alone had only a minor effect on endoreplication (Fig 4F). Nevertheless, we were able to show that overexpressing *snail* inhibited endoreplication cell-autonomously in the PG. Taken together, our data demonstrated that *snail* levels in the PG need to be tightly controlled around the time of CW attainment for endocycle progression.

### Snail activity in the PG around the CW period is essential for endocycle progression, CW attainment, and the onset of metamorphosis

We have established that *snail* has a function in controlling endoreplication in the PG. We therefore reasoned that if *snail* functions solely in regulating endoreplication, we would observe a rescue of PG>*snail*-RNAi with ectopic expression of *CycE*. This is because CycE promotes entry into S phase [62,69] and was successfully used to rescue TOR-deficient cells that had arrested endoreplication in PG cells [23]. However, promoting S-phase entry by overexpressing *CycE*-cDNA did not rescue the L3 arrest phenotype in PG>*snail*-RNAi animals (S4 Fig). These results suggested that *snail* has more complex roles in the PG than simply controlling ecdysone biosynthesis and endocycling.

**Fig 5:**
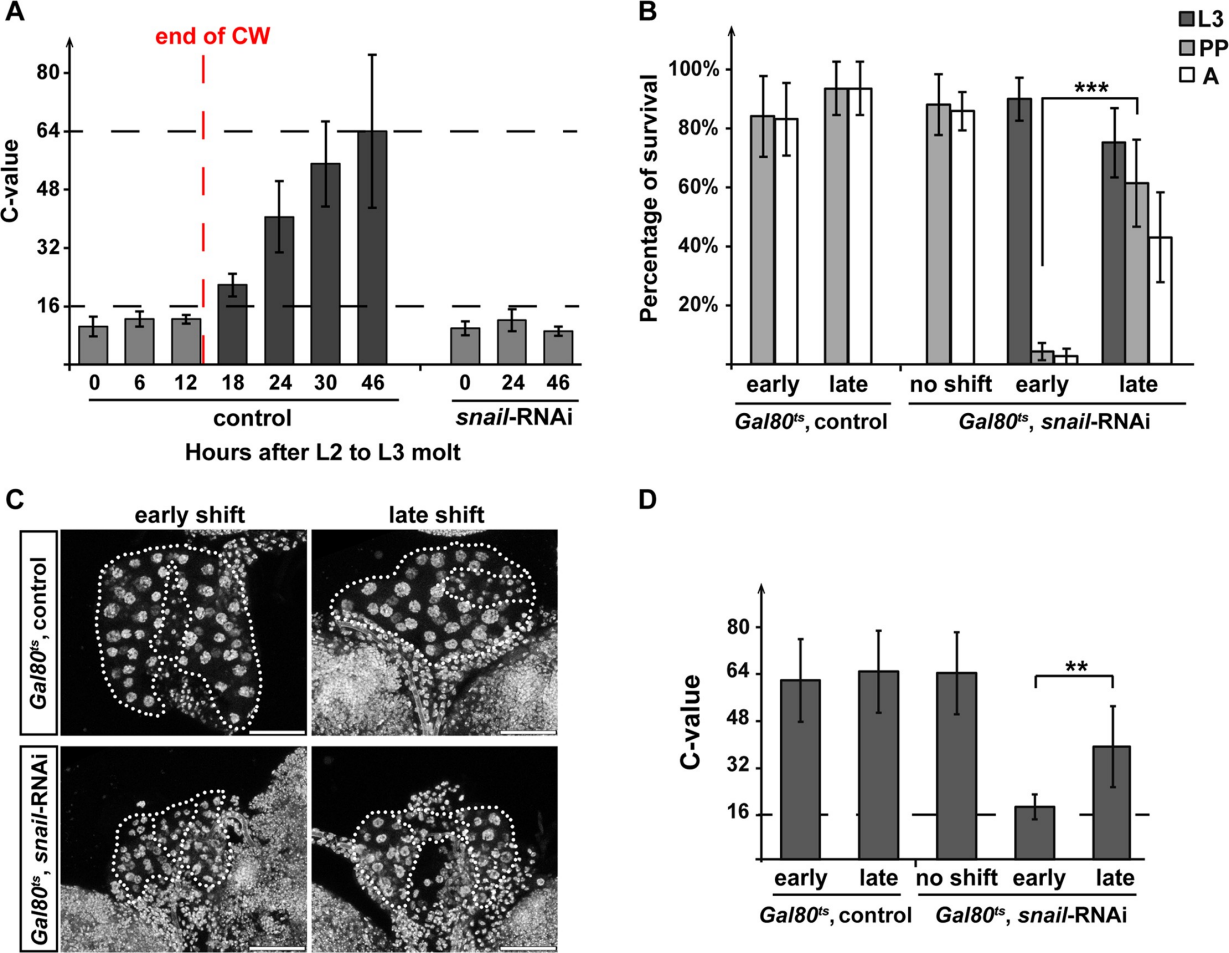
*snail* function is required in the PG at the time of CW attainment for endoreplication. **(A)** Bar graph showing the C-value of the PG cell at detailed time points after the L2/L3. Relative DNA content per PG cell was determined by the DAPI intensity in the PG normalized to the mean DAPI intensity of the brain hemisphere. Average DNA content in controls at 46 hr L3 is normalized to 64C. The dotted line marks the threshold of DNA content per PG cell before the CW checkpoint (gray bars are all below the threshold). After 12 hr L3 (post-CW) the DNA content in controls increased beyond the threshold (black bars). End of CW: developmental time when the entire population attained CW (also minimal viable weight). Error bar represents standard deviation. ctrl.: *UAS*-*Dicer2*; *phm22*-*Gal4*>*UAS*-*EGFP* and *snail*-RNAi: *UAS*-*Dicer2*; *phm22*-*Gal4*>*UAS*-*snail-*RNAi; *UAS*-*EGFP*. **(B)** Bar graph showing percentages of animals survived to indicated stages in various temperature-shift scheme. no shift: continuous 18°C (with no transgene induction). early: animals were shifted to 29°C at 144 hr AED to activate the RNAi construct. late: animals were shifted to 29°C at 168 hr AED to activate the RNAi construct. **(C)** Expression of *snail*-RNAi before CW attainment causes arrest of DNA content increase. Maximal projection of Z-stack confocal images showing the size of the PG nuclei in various temperature-shift schemes described in panel (B). RGs were dissected 3 d after the temperature shift when control genotype reached the end of the larval stage. Tissues were stained with DAPI. Scale bars, 50 μm. **(D)** C-value of the PG cells calculated using the summation of the Z-stack images shown in panel (C). Average DNA content at the end of the larval stage in late-shift control samples is normalized to 64C. The white dotted line marks the boundary of the PG and CA. (B-D) *Gal80*^*ts*^, control: *snail*-RNAi; *tub-Gal80*^*ts*^>*w*^*1118*^. *Gal80*^*ts*^, *snail*-RNAi: *snail*-RNAi; *tub-Gal80*^*ts*^>*phm22*-Gal4. Underlying data for this figure can be found in S1 Data. A, adults; AED, after egg deposition; CA, corpus allatum; C-value; DNA content value; CW, critical weight; Gal80^ts^, Gal80 temperature-sensitive allele; L2, second instar; L3, third instar; PG, prothoracic gland; PP, pupae; RG, ring gland; RNAi, RNA interference.

A previous study proposed that endocycle progression in the PG is an intrinsic timer for the transition to maturation (onset of metamorphosis) and is strongly correlated with CW attainment [23]. Specifically, one round of endoreplication will occur around the time of CW attainment, allowing the C-value to increase beyond 16 in nonstarved conditions [23]. In contrast, larvae starved before CW displayed PG cells with DNA content not exceeding 16C [23]. In addition, larvae starved in this manner arrested development and failed to pupariate [23] (S5A Fig). We hypothesized that when *snail* function is disrupted in the PG, the C-value of PG cells also stopped at 16, which in turn is interpreted by larvae that they have not attained CW. Therefore, we would predict that Snail-depleted animals will keep feeding and never initiate metamorphosis because of a failure in up-regulating ecdysone biosynthesis. To test this hypothesis, we measured the DNA content of PG cells for both controls and *snail*-RNAi animals throughout the L3 stage, when the enhanced GFP (EGFP) marks PG cells. We first established that, in our hands, CW is attained around 9–10 hr L3 in control genotypes using the criteria of minimal viable weight (the time when >50% of the larvae can pupariate when they are starved) (S5B Fig). By 13 hr L3, 100% of the larvae have attained CW. When we monitored C-values in control larval during and after the CW checkpoint, we noticed DNA content higher than 16C directly after the attainment of CW (Fig 5A), consistent with earlier findings [23]. Noticeably, the C-value remained constant in the first 12 hr of L3 (Fig 5A), and along with the low DNA replication activities observed in late L2 (20 hr and 22 hr L2) (Fig 3D), we concluded that endocycle progression in the PG is slowed between two *snail* (as well as endoreplication) peaks. We hence refer to this period of development as “endoreplication pausing.” As such, PGs escape from endoreplication pausing at CW attainment, thus resulting in a C-value > 16. In *snail*-RNAi PGs, however, C-values did not exceed 16, even at later time points when controls had pupariated, indicating that escape from endoreplication pausing around the CW checkpoint is *snail*-dependent.

To further examine the role of *snail* around the time of CW attainment, we disrupted *snail* function directly before and after the CW window. To do this, we used the Gal80^ts^ system to exert temporal control [57]. Since this approach requires a shift from 18°C to 29°C, we needed to determine when CW was attained at 18°C, which turned out to be between 168 hr AED (about 21% of larvae had reached CW) and 175 hr AED (about 54% of larvae had reached CW) (S5C Fig). We reasoned that because of the delay of activating the *snail*-RNAi transgene at the temperature shift, a shift to 29°C at 144 hr (“early shift”) would ensure that *snail*-RNAi was effective before the CW checkpoint (100% of larvae have not reached CW, S5C Fig), whereas a shift to 29°C at 168 hr (“late shift”) would ensure that *snail*-RNAi was effective right after CW was obtained. We found that the activation of *snail*-RNAi at 144 hr AED (early shift) or earlier caused L3 arrest. In contrast, the “late shift” induction of *snail*-RNAi resulted in about 60% pupal and about 45% adult survival (Fig 5B). These data clearly confirmed a critical role for *snail* during the CW checkpoint. Next, we tested whether C-values differed between early and late activation of *snail*-RNAi. Indeed, in “late-shift” larvae, the C-value of PG cells reached >32 at the end of L3, whereas in “early-shift” larvae, C-values did not exceed 16 (Fig 5C and 5D). These results demonstrated that Snail is required for exceeding 16C in PG cells (demarcating the end of endocycle pausing), which allows PG cells to exit the CW checkpoint and proceed to metamorphosis.

### *snail* expression in the PG is nutrient-dependent around the time of CW attainment

**Fig 6:**
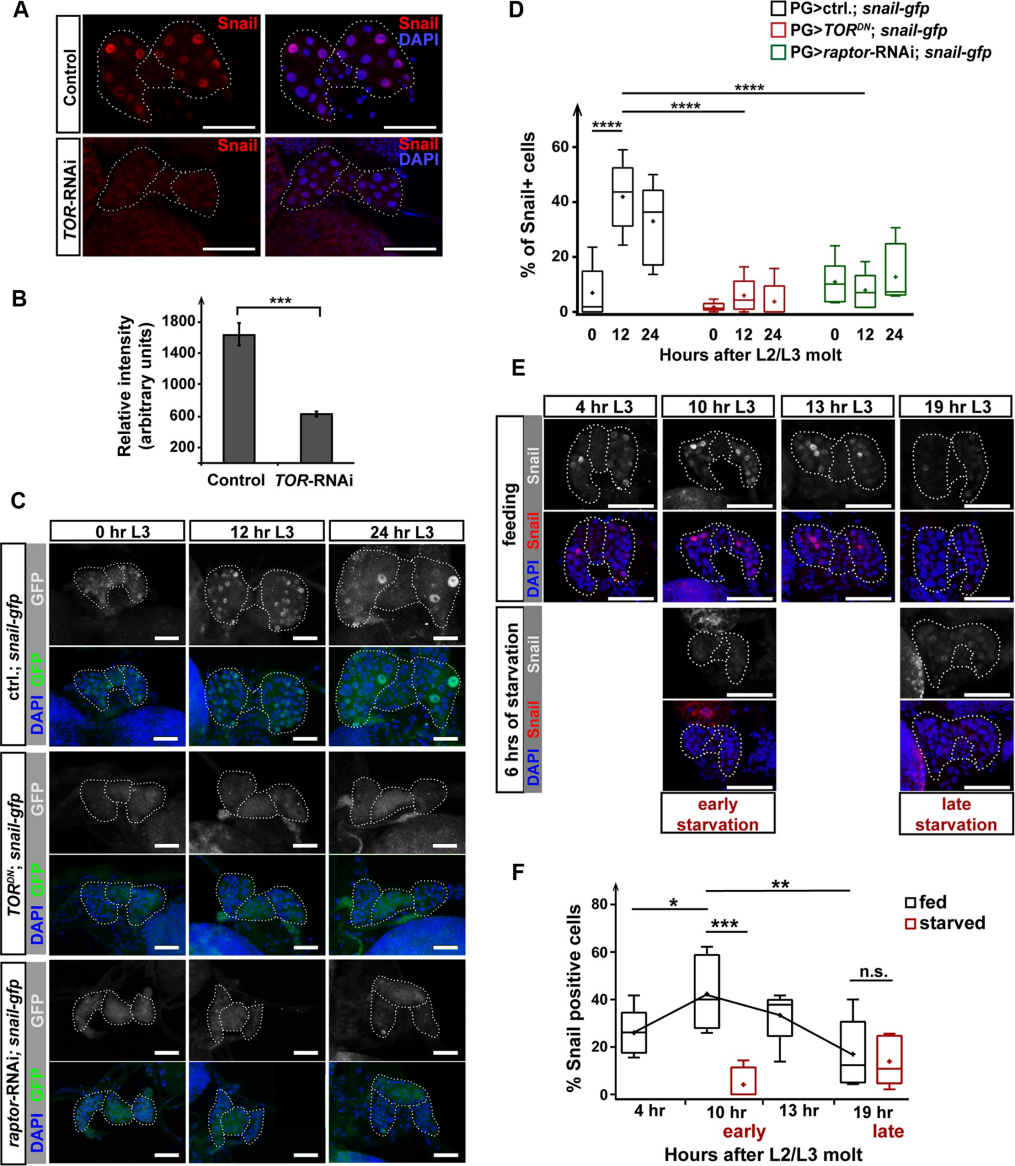
Snail levels in the PG are dependent on TOR signaling and nutrient status. **(A)** TOR-RNAi affected Snail levels in the PG. Tissues were stained with anti-Snail antibody as well as DAPI. The figures show single-plane confocal images. The average Snail fluorescence intensity in nuclei was quantified in **(B)**. Error bars represent standard error. ***p < 0.001; (Student *t* test). control: *phm22*>*w*^*1118*^. *TOR*-RNAi: *phm22*>*TOR*-RNAi. **(C)** Immunofluorescent images showing GFP-tagged Snail using the *snail-gfp* line in control (*phm22-Gal4*/+; *snail*-*gfp*), *TOR*^*DN*^ (*phm22-Gal4*/*UAS*-*TOR*^*DN*^; *snail*-*gfp*), and *raptor*-RNAi (*phm22-Gal4*/*UAS*-*raptor*-RNAi; *snail*-*gfp*) RGs. **(D)** Box plot represents the percentage of Snail-positive nuclei in the PG quantified from the images in panel (C). **(E)** Maximal projection of confocal images showing the Snail protein distribution in the PG under fed and starved conditions. *w*^*1118*^ larvae were either fed with standard cornmeal-based medium or starved on 2% agar. Tissues were stained with the anti-Snail antibody and DAPI. early starvation: starvation started before CW attainment (at 4 hr L3). late starvation: starvation occurred after CW attainment (at 13 hr L3). **(F)** Box plot shows the percentage of Snail-positive cells in the PG quantified from the results in panel (E). The average values of controls at each time point are connected by the black line. early: early starvation. late: late starvation. (D and F) *p < 0.05, **p < 0.01, ***p < 0.001, ****p < 0.0001 (two-way ANOVA). (A, C, and E) PG and CA are outlined by a white dotted line. Scale bars: 50 μm. Underlying data for this figure can be found in S1 Data. CA, corpus allatum; CW, critical weight; L2, second instar; L3, third instar; n.s., not significant; PG, prothoracic gland; RNAi, RNA interference; TOR, target of rapamycin; *TOR*^*DN*^, dominant-negative form of TOR.

Endocycling around the time of CW attainment is nutrient-dependent [23]. TOR is a key component of nutrient-sensing machinery in eukaryotes [70–72]. When TOR function is disrupted in the PG before the end of the CW window, endoreplication of PG cells is blocked at 16C, which, incidentally, is also observed with PG-specific *snail*-RNAi. However, the endocycle will not be affected if TOR function is impaired after CW attainment, suggesting TOR is required for promoting the endocycle during the CW window [23]. TOR is also known to regulate ecdysone biosynthesis in the PG [18]. Therefore, we hypothesized that *snail* might act in the same pathway as TOR and that TOR could act upstream of *snail*. To test this, we examined the presence of Snail in *TOR*-RNAi PGs via immunofluorescence at the L2 to L3 molt. We chose the L2/L3 molt to ensure that the control (PG>*w*^*1118*^) and PG>*TOR*-RNAi populations were developmentally synchronized, since PG>*TOR*-RNAi larvae later arrest in the L3 stage [18]. These immunostains revealed that Snail levels were significantly reduced in the *TOR*-RNAi PG when compared to controls (Fig 6A). Since Snail was only present in a few cells within the PG, we selected three Snail-positive nuclei from individual PGs to quantify the average Snail intensity. This approach showed that the difference in immunofluorescent signals between *TOR*-RNAi and controls was highly significant (Fig 6B).

We obtained similar results when we ectopically expressed the toxic extended domain (TED) of TOR (UAS-TOR.TED), which acts as dominant-negative (TOR^DN^), in a *snail*-GFP background. We examined Snail levels by GFP staining at three time points, namely 0 hr L3 (time before CW checkpoint), 12 hr L3 (close to the end of CW checkpoint), and 24 hr L3 (when the entire population attained CW). We then quantified the percentage of Snail-positive nuclei per PG. In controls, we again observed an increase in Snail-positive cells in 12-hr-old L3 PGs (Fig 6D). In contrast, the percentage of Snail-positive cells remained low at all three time points in PG>*TOR*^*DN*^ (Fig 6C and 6D). Moreover, TOR forms two distinct protein complexes, TORC1 and TORC2 [73], among which only the TORC1 complex was shown to couple the nutrient-dependent endocycle progression to ecdysone biosynthesis [23]. Therefore, we also tested the effect of loss of *raptor* function on Snail levels, since Raptor is a key component of the TORC1 complex [74]. Consistent with the TOR^DN^ results, the number of Snail-positive cells were also reduced in *raptor*-RNAi PGs (Fig 6C and 6D).

The IIS and TOR pathways are interconnected at multiple steps in *Drosophila* [20,75,76], and IIS signaling is also at the core of the nutrient-sensing system that couples growth to nutritional conditions [70]. Therefore, we tested whether IIS signaling was required of *snail* expression. Disrupting IIS signaling using the PG driver (*phm22*-*Gal4*) with a range of different transgenes typically arrested development in the L2 stage, which is why we employed a weaker RG driver, *P0206-Gal4*, to obtain animals that reach the L2/L3 molt. We disrupted IIS signaling in three ways: (1) expressing a dominant-negative form of the insulin receptor (*InR*^*DN*^); (2) expressing *Pten*, a negative regulator of the pathway; (3) knocking down *Akt1*, a player further downstream of the pathway. Despite this triple approach, we only observed reduced *snail* expression in *Pten* overexpression (S6A and S6B Fig). The P0206 driver also carries the *UAS-EGPF*:*mCD8* construct, which results in the labeling of the cell membrane with GFP, allowing us to assess cell size. Using this strategy, we found that the size of PG cells in *P0206*>*Akt1*-RNAi were heterogeneous. Importantly, there appeared to be no correlation between nuclear Snail levels and cell size (S6A Fig), indicating that reduced *snail* levels in *TOR* loss of function as well as *Pten* overexpression was a specific effect and not a secondary consequence of reduced cell size. It was still possible that IIS signaling was not disrupted to the point that one could observe an effect on *snail* expression with the weak *P0206* driver in *InR*^*DN*^ animals. Therefore, we knocked down *p110* (the catalytic subunit of PI3K encoded by *Pi3K92E* in *Drosophila*), a positive effector of the pathway using the stronger PG driver (*phm22-Gal4*), in which case we were able to obtain 0 hr L3 animals. The *PG>Pi3K*-RNAi caused inconsistent results in which some of the PG cells had no detectable *snail* expression, whereas other cells appeared normal (S6C Fig). Taken together, our results showed that proper *snail* expression in the PG is primarily dependent on TOR function but, to a lesser degree, was also affected by IIS signaling. TOR activity is thought to be more critical for regulating the size of endoreplicating tissues compared to IIS signaling [77], which may explain why *snail* function appears to be more sensitive to the disruption of TOR function than interfering with IIS signaling. Taken together, *snail* appears to act downstream of TOR in regulating the nutrient-dependent endocycle progression around the time of CW checkpoint.

**Fig 7:**
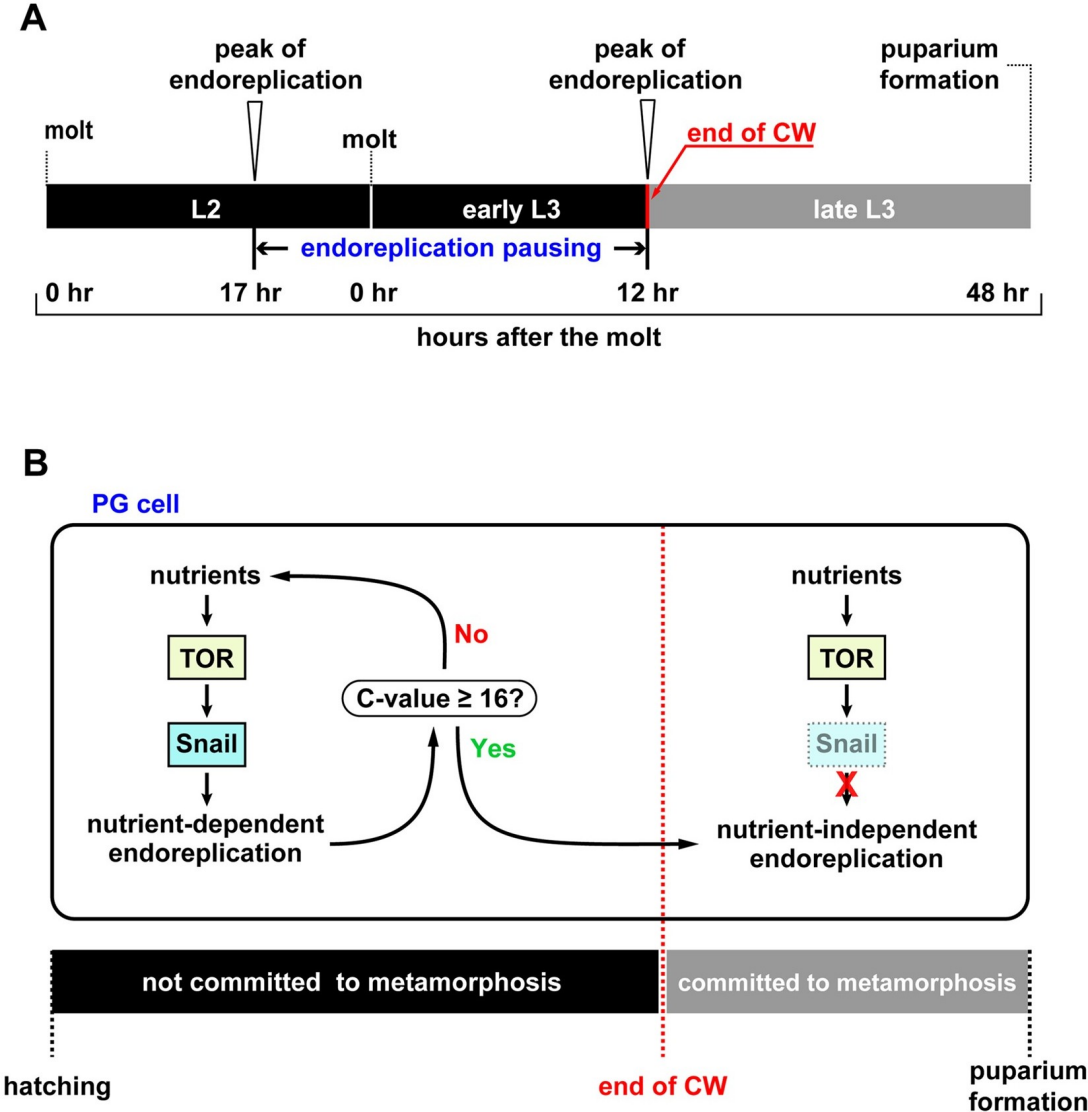
Model of Snail function in the PG. **(A)** Two waves of endoreplication in the PG were observed in late L2 and early L3, flanking a development stage with low endocycle activity (“endoreplication pausing”). Endoreplication pausing ends at the time of CW attainment. **(B)** Snail function in the PG in regulating endoreplication around the time of the CW checkpoint. Before the CW attainment, animals’ commitment to metamorphosis is dependent on nutrient availability. Nutrient conditions mediated by the TOR signaling control the levels of Snail to regulate endoreplication. During the CW time window, the size and the nutrient status of the animals are molecularly assessed via the C-value of the PG. Once mandatory rounds of endoreplication have occurred in the PG, animals pass CW checkpoint [23]. Once the CW checkpoint is fulfilled, animals are committed to metamorphosis. Subsequently, Snail levels decline so that nutritional inputs are no longer relevant and the endocycle progression is no longer dependent on nutrients after the CW checkpoint. C-value; DNA content value; CW, critical weight; L2, second instar; L3, third instar; PG, prothoracic gland; TOR, target of rapamycin.

Lastly, we examined whether the expression of *snail* around the time of the CW checkpoint was directly affected by nutrient availability. To test this, we performed two starvation schemes on control larvae: (1) animals were synchronized at 4 hr L3 (before the CW checkpoint) and then starved for 6 hr (*“*early starvation”); (2) larvae were starved at 13 hr L3 (after the CW checkpoint) for 6 hr (*“*late starvation”). We showed by immunofluorescent staining that the early starvation procedure abolished *snail* expression, whereas the developmentally matched feeding counterpart (reached 10 hr L3 developmentally) had a high percentage of Snail-positive cells (Fig 6E and 6F). The “late-starvation” regimen also reduced *snail* expression; however, in appropriately age-matched feeding larvae, *snail* expression was already declining as well (Fig 6E and 6F). Taken together, these results demonstrated that *snail* expression is strongly dependent on nutrient conditions around the time of the CW checkpoint but not after the CW is attained, since the expression declines after the CW attainment regardless of the nutrient conditions (Fig 7). Hence, the *snail* expression profile is consistent with the idea that it acts as a molecular switch before and after CW attainment as well as an intrinsic timer for the timing of onset of metamorphosis.

## Discussion

### Snail as a regulator of ecdysone production in the PG

In this study, we demonstrated that *snail* is dynamically expressed in the PG, the main endocrine organ for ecdysone biosynthesis during larval stage. Snail is required for the expression of the six main ecdysone biosynthetic genes, consistent with a low ecdysteroid titer in the *snail*-RNAi larvae (Fig 2C). Consequently, PG-specific loss-of-function animals displayed developmental arrest, since ecdysone triggers each developmental transition (Fig 1B). However, high levels of *snail* in the PG also appeared to block ecdysone biosynthesis, evidenced by the fact that overexpressing *snail* in the PG caused developmental arrest (Fig 2D). Also, the expression of *dib*, *sro*, *phm*, and *sad* was significantly suppressed by PG-specific *snail* overexpression (Fig 2F). These results suggested that Snail levels are tightly controlled in the PG to ensure appropriate regulation of the Halloween genes. It is possible that Snail impinges directly and indirectly on ecdysone biosynthesis, as some of the Halloween genes may constitute direct targets of this transcription factor. This hypothesis would also explain why *snail* levels drastically decline during the second half of the L3 stage and become almost undetectable at the end of this stage (Fig 1A; S1 Fig), since a major ecdysone peak needs to occur at this time to initiate the onset of metamorphosis.

Several zinc finger transcription factors have been shown to specifically regulate the expression of single or a group of ecdysone biosynthetic genes in the PG [78–80]. Further studies are required to assess whether Snail directly controls the transcription of any of the six major ecdysone biosynthetic genes. Snail is a classic transcriptional repressor [81–83]. However, a recent study also showed that it can potentiate gene activation. Whether Snail represses or activates gene transcription likely depends on promoter/enhancer context, such as the co-binding of Twist at enhancer elements of the target genes, at least during embryogenesis [84]. Typically, the spatiotemporal regulation of target genes is achieved by the coordinate action of multiple transcription factors binding to *cis*-regulatory modules. This may explain why both the loss of *snail* and overexpression of *snail* reduced the transcript levels of the ecdysone biosynthetic genes.

According to the chromatin immunoprecipitation (ChIP)-on-chip results released by Berkeley *Drosophila* Transcription Network Project (BDTNP), Snail binds near the *phm* and *sad* genes during embryonic stage 5 (S5 Table), raising the possibility that it regulates the transcription of these two ecdysone biosynthetic genes in the PG. We then followed up by carrying out an in silico search for the potential Snail binding sites in the six major ecdysone biosynthetic genes based on the consensus Snail binding motifs from multiple databases [85] using the web server IN-silico SEarch for Co-occurring Transcription factors (INSECT 2.0). Using INSECT 2.0, the sequence within 2 kb upstream and 1 kb downstream from the transcription start site was examined for transcription binding sites. Interestingly, all six ecdysteroidogenic enzyme genes have predicted Snail binding sequences (S5 Table). Some of the predicted binding sites in *sro*, *phm*, *dib*, and *sad* (but not in *nvd* and *spok*) are conserved among several other *Drosophila* species, suggesting that they might be functionally important and represent bona fide Snail binding sites (S5 Table). This is consistent with the finding that the expression of *sro*, *phm*, *dib*, and *sad* was repressed (but not for *nvd*, *spok*) in *snail*-overexpression RGs (Fig 2E). A possible explanation is that high occupancy of Snail on these four genes (*sro*, *phm*, *dib*, and *sad*) inhibited appropriate cofactor binding, interfering with normal *sro*, *phm*, *dib*, and *sad* expression. As such, we predict that *sro*, *phm*, *dib*, and *sad* are bound and regulated by Snail, but whether they are repressed or activated by Snail is context-dependent. Therefore, it appears possible that four of the ecdysone biosynthetic genes (*sro*, *phm*, *dib*, and *sad*) are directly regulated by Snail, which can be tested by PG-specific ChIP analysis in the future.

### Snail as a novel regulator of endocycling in the PG

We also showed that Snail is a novel regulator of endoreplication in the PG. Snail has a peculiar mosaic distribution in the PG, which is strikingly similar to the pattern of the EdU-labeled S-phase cells. The percentage of Snail-positive cells correlated well with the percentage of the S-phase cells at different developmental time points (Figs 1A, 3D and 3E; S1 Fig). Moreover, loss of Snail function resulted in endocycle arrest in the PG (Fig 3A and 3D). However, we have not yet identified the endocycle-related genes that are possibly regulated by Snail in the PG via our RNA-Seq analysis. One possible reason is that the time point (24 hr L3) we used to collect samples for the RNA-Seq analysis was way after CW checkpoint when endoreplication in the PG was already neither nutrient- nor *snail*-dependent. Therefore, we may not see a significant effect of *snail*-RNAi on endocycle-regulated genes. Our in silico search using INSECT 2.0 revealed that one of the key genes that regulates the S phase of the cell cycle, *dup* [86], contains nine potential Snail binding sites (S6 Table). Four out of nine predicted binding sites were found in almost all the 12 *Drosophila* species, suggesting that they are functionally important. The C*ycE* gene appears to harbor Snail binding sites in the intronic region, and proper *CycE* expression pattern in the embryo stage is dependent on Snail [84].

Cell proliferation or cell survival are obviously important aspects of cell cycle regulation [87,88]. We showed here that the loss of *snail* function resulted in a reduced cell number in the PG, whereas overexpressing *snail* increased the cell number in the PG. Decreased cell numbers in the PG were also observed in PG*>CycE*-RNAi and PG-specific knockdown of other endocycle regulators, whereas overexpressing *CycE* in the PG resulted in increased cell number (S3 Fig) [23]. These changes in cell numbers are possibly one of the consequences of misregulated cell cycle processes caused by impaired Snail function. In line with the changes in cell numbers, we noticed that two apoptotic genes, *reaper* (*rpr*) and *head involution defective* (*hid*), were 3.6-fold (*p* = 0.0289) and 5.5-fold (*p* = 0.011) down-regulated in RG samples isolated from hs>*snail*-cDNA larvae. This was based on the RNA-Seq results, and we also validated these results using qPCR analysis (S3 Table and S7 Fig). Interestingly, the vertebrate Snail family proteins Snail1 and Snail2 regulate cell survival and apoptotic genes [89–92], and a recent study in *Drosophila* showed that *snail* specifically modulates the type of cell death mediated by c-Jun N-terminal Kinase [93]. Hence, Snail seems to partake in certain cell cycle events that are vital for normal growth of the endoreplicating tissue PG (endoreplication) as well as for cell death/survival.

### Snail as a candidate of CW determinant

The timing of CW attainment is strongly correlated with the time at which PG cells have completed three mandatory rounds of endoreplication, suggesting that DNA amounts exceeding 16C could act as a molecular readout that occur when animals have passed the CW checkpoint [23]. Furthermore, endoreplication in the PG around the CW checkpoint is nutrient-dependent and requires TOR function, but not after the checkpoint is fulfilled [23]. Despite the coupling of CW attainment and TOR-mediated endoreplication in the PG, we still lack an understanding of the actual mechanism that underlies the switch-like nature of the CW checkpoint. A transcription factor would constitute a likely candidate for this process, since drastic transcriptional changes may, at least in part, account for the switch-like nature of CW attainment. In this study, we showed that two rounds of endocycling, at late L2 (17hr L2) and 12 hr L3, flank a period of low endoreplication activity, which we termed as endoreplication pausing (Figs 3D and 7A). The escape from endoreplication pausing happens at CW attainment, after which PG cells will continue to increase their DNA content beyond 16C. Interestingly, knocking down *snail* before CW attainment affected the onset of metamorphosis, and the PG endocycle was arrested at 16C (failure to escape from endoreplication pausing). In contrast, the loss of *snail* after CW attainment no longer blocked pupariation, and the C-value was able to increase beyond 16 (Fig 5B–5D). We also demonstrated that *snail* expression in the PG is dependent on TOR function, as well as nutrient conditions (Fig 7B). Interestingly, Snail levels are more sensitive to the nutrient status before CW attainment than after CW attainment (Fig 6E and 6F). These results strongly suggest that Snail acts downstream of TOR and is part of the molecular mechanism by which the CW attainment time window is determined (Fig 7).

### Snail as a putative PG identity maintenance protein

Snail is one of the classic embryonic patterning genes identified in Heidelberg screen [94,95]. One study showed that Snail is essential for specifying the endocrine precursor cell fate that gives rise to the RG via regulating the epithelial to mesenchymal transition [48]. Specifically, the endocrine and the tracheal primordia cells originate from common precursor cells that characteristically express the transcription factor *ventral veins lacking* (*vvl*), loss of which resulted in the degradation of these common precursor cells. From these common precursor cells, endocrine (RG) cell fate was specified by the subsequent activation of Snail. Intriguingly, *vvl* is also expressed later in the larval PG cells, which constitutes the biggest component of the RG. *vvl* regulates the expression of ecdysone biosynthetic genes in the PG during larval development [54,96]. More interestingly, *vvl* function in the PG is also required for cell growth (probably endoreplication, since endoreplication is typically the main way of cell growth in an endoreplicating tissue). Lastly, *vvl* function is essential in the PG in the early L3 stage (presumably around the time of CW attainment) but is no longer important in later L3 (after CW checkpoint). These findings are strikingly similar to our observations. Thus, it appears plausible that both Vvl and Snail are important for RG fate determination and that both are later required during larval stages for ecdysone production, endoreplication, and metamorphosis. This raises the possibility that the same program that is used to specify the cell fate of the embryonic RG is reused in the larval PG to maintain PG cell identity and to regulate the CW checkpoint.

## Materials and methods

### Fly stocks and husbandry

Flies were maintained on a standard agar-cornmeal medium at 25°C. *phm22*-*Gal4* (on the third chromosome), *phm22*-*Gal4/CyO* (on the second chromosome), and *spok*^*GS*^-*Gal4* were obtained from Dr. Michael B. O’Connor’s lab. *UAS-snail-*RNAi (#50003) was from the VDRC. *UAS-Dicer2*-cDNA (#24650), *UAS-TOR*^*DN*^ (#7013), *UAS-TOR*-RNAi (#34639), *UAS*-*raptor*-RNAi (#41912), *UAS*-*Pi3k92E*-RNAi (#27690), *UAS*-*Akt1*-RNAi (#31701), and *UAS-CycE*-cDNA(#30725) were ordered from Bloomington stock center. *UAS*-*snail*-cDNA (#109121) were obtained from Tokyo Stock Center and *UAS*-*Pten*-cDNA from FlyORF (#F001338). *hsFLP*; *tub*-*FRT-CD2- FRT-Gal4*, *UAS-GFP* is a kind gift from Dr. Hwei-Jan Hsu [68]. The P[acman] *snail-*GFP line with an in-frame C-terminal GFP-cDNA fusing to the *snail* locus was a kind gift from Dr. Angelike Stathopoulos [50].

### Generation of *sna*-Enh conditional allele with CRISPR/Cas9-mediated HDR

The *sna*-Enh^FRT^ allele was generated by inserting two FRT sites at 3,247 bp and 6,421 bp upstream of the transcription start site of *snail*, thus flanking the *snail* enhancer region (−5,967 to −4,085 from the transcription start site) important for *snail* expression in the RG with FRTs [48] (Fig 1D). This specific modification was achieved via CRISPR/Cas9-mediated homology directed repair (HDR) using two site-specific guide RNAs (gRNAs) and a donor template [97]. Two gRNAs (5′-ATTTAGGAGACACGTGTAAT-3′ and 5′-GGCGTCGCTCGATATGTATA-3′) were cloned into pCFD4 following the protocol developed by Port et al [98]. Primers for dual gRNA cloning are listed in S7 Table. The donor template for HDR was cloned using pHD-DsRed-attP [97], and details on cloning are described in S1 Text. Injection of the dual gRNA vector and the donor template into *nos*-Cas9 embryos (G0) were done by Genetivision (Houston, TX, United States). Positive transformants were identified by screening for dsRed in the F1. Finally, *sna*-Enh^FRT^/*sna*-Enh^FRT^; *phm22*-Gal4/UAS-FLP were generated by genetic crosses to flip out the enhancer sequence in PG cells (*sna*-Enh^ΔRG^, Fig 1D).

### Next-generation RNA-Seq analysis

Sixty RGs were dissected for each sample at 24 hr L3. Each condition was tested with two biological replicates. RGs were dissected from the larvae at desired time points in ice-cold 1x PBS buffer and put immediately into TRizol reagent (Ambion, Life Technologies) in a 1.5-ml Eppendorf tube. The tube was then flash-frozen in liquid nitrogen for short-term storage. Upon total RNA extraction, tissues were homogenized with a pestle presoaked in 1% SDS. RNA was then isolated by phenol-chloroform phase separation; the aqueous phase (contains the RNA) was subjected to the QIAGEN RNeasy spin column (RNeasy mini kit) for further purification following the manufacturer’s instructions. The DNAse (supplied by the RNAeasy mini kit) digestion step was administered during the RNA extraction according to the manufacturer’s instructions to eliminate the genomic DNA. The purity of the RNA was assessed by the ratio of the absorbance at 260 nm and 280 nm using NanoDrop ND-1000. Samples with an A260/A280 value below 1.8 were discarded. In addition, the integrity of the RNA samples was then assessed using Agilent RNA 6000 Nano chip (RNA 6000 Nano kit) run on a 2100 Bioanalyzer Instrument. RNA concentration was measured using the Qubit RNA HS Assay Kit (Thermo Fisher Scientific #Q32852). In this study, 30 ng of total RNA was used for each sample. cDNA libraries were constructed using the Ovation Universal RNA-Seq kit for *Drosophila* (NuGEN #0350) following the manufacturer’s instructions. The final library for each sample was analyzed by an Agilent Bioanalyzer DNA1000 chip, and the software can estimate the average of the library size distribution, which ideally should be around 275 bp. Then, the libraries were quantified using Qubit dsDNA BR Assay Kit (Thermo Fisher Scientific #Q32850). Next-generation sequencing was performed by Delta Genomics (Edmonton, Alberta, Canada) on an Illumina Hi-Seq 2500 platform. Raw data were analyzed by Arraystar 4.0 (DNAstar) to create individual files for each sample. Data were analyzed with Arraystar 4.0 (DNAstar) as well as Microsoft Access. GO statistics was performed with DAVID and STRING [59,60].

### 20E rescue experiments

A 20E stock solution at a concentration of 10 mg/ml was made in ethanol. The 20E stock solution (0.5 ml) was then added to every 15-ml agar-cornmeal medium before it solidified, which resulted in a final concentration of 333 μg/ml for 20E (with 3.3% ethanol). The control food was prepared similarly with only 3.3% ethanol. Fifty larvae were transferred to each vial and allowed to develop at 25°C, after which the phenotype of the larvae was scored.

### Quantitative RT-PCR

Thirty-five to 50 RGs were used for each sample. The RG RNA was extracted as described for RNA-Seq analysis. For cDNA synthesis, 50–100 ng of total RNA was used with the High-Capacity cDNA Reverse Transcription Kit (Applied Biosystems, ABI #4368814) following manufacturer’s instructions. The qPCR reactions were set up using the 2x SYBR Fast qPCR Master Mix (Kapa biosystem #KK4601) with a primer concentration of 800 nM and 2.5 μl of transcribed cDNA in triplicate. The reactions were performed on a QuantStudio 6 Flex Real-Time PCR System using the comparative C_T_ (ΔΔC_T_) mode with the following thermocycling parameters: (step 1) 95°C for 20 s; (step 2) 95°C for 1 s; and (step 3) 60°C for 20 s. Steps 2 and 3 were performed for a total of 40 cycles. All primer sequences can be found in S7 Table.

### 20E measurements

Ecdysteroid titers of whole larvae were measured following the manufacturer’s instructions of the 20-Hydroxyecdysone Enzyme Immunoassay kit (Bertin Pharma #A05120.96 wells). In brief, 35–45 larvae were collected per sample and homogenized in 400 μl of methanol with a motorized pestle to extract the ecdysteroids. Supernatants were transferred into a new tube after centrifuging at the maximal speed (13.2 × 1,000 rpm). Another round of extraction was done with the old lysate in 400 μl of methanol followed by one more extraction with 400 μl of ethanol. All the extracts were pooled together (1.2 ml in total) and dried by Speed-Vacuum. Next, the samples were redissolved in 110 μl of EIA buffer (provided by the kit) for 2 hr at room temperature (RT) or overnight at 4°C. The 96-well plate was prewashed with wash buffer, after which 50 μl per sample, as well as controls, were loaded onto the plate in duplicate. The plate was incubated at 4°C overnight, and the plate was then emptied and washed with 300 μl wash buffer. In the end, the assay was developed with 200 μl of Ellman's reagent and incubated in the dark at RT on the shaker. After 75 min, results were obtained at a wavelength of 410 nm using the Synergy H1 microplate reader (BioTek).

### Immunofluorescence with larval tissues

Larvae were cut at the one-third portion toward the head with a pair of Dumont #5 forceps (Fine Science Tools #11252–40) in 1x PBS. The cuticle was then turned inside-out to expose the tissues. RGs, still attached to the brain and the brain to the mouth hook, were transferred into 4% paraformaldehyde (PFA) (Electron Microscopy Sciences, #RT15710) in a 0.6-ml reaction tube. Tissues were fixed for 22 min at RT and then washed three times with PBS containing 0.3% Triton-X 100 (PBST) for 15 min each, followed by blocking with 5% normal goat serum (NGS) in PBST for 30 min at RT. Tissues were incubated with primary antibodies either at 4°C overnight or at RT for 4 hr followed by three washes (with PBST) and then incubated in the secondary antibody for 1 hr at RT. Tissues were washed three times (with PBST), and DAPI at a concentration of 1 μg/ml was added at the last wash to stain nuclei. Finally, the RGs were mounted onto the slides in VECTASHIELD Antifade Mounting Medium. Rabbit anti-Snail is a kind gift from Dr. Zeitlinger and was used at a 1:600 dilution. Rabbit anti-GFP was used 1:20 (Thermo Fisher Scientific, #G10362). Secondary antibodies (Goat anti-Rabbit Alexa Fluor 488, anti-Rabbit Alexa Fluor 555, and anti-mouse Alexa Fluor 555) were used at a 1:500 dilution. Images were captured on a Nikon C2+ confocal microscope.

### EdU incorporation assays

EdU incorporation assays were carried out using Click-iT EdU 555 Imaging Kit (Life Technologies #C10338). Briefly, larvae were dissected in 1x Ringer’s solution and were incubated for 30 min at RT with 10 μM EdU. Tissues were then fixed in 4% PFA in PBS for 25 min followed by two brief washes in 0.3% PBST and then washed again twice for 20 min each in 0.3% PBST. Blocking was performed with 1% bovine serum albumin (BSA) in 0.3% PBST for 30 min, and tissues were then incubated with Click-iT reaction cocktail (prepared according to manufacturer’s instructions) for 30 min at RT. Tissues were washed in 0.3% PBST and incubated with Hoechst 33342 at a 1:1,500 dilution in 0.3% PBST for 5 min.

### Generation of *snail*-overexpressing clones by FLP-out system

Flies of *y*, *w*, *hsp70-FLP*; *tub*-*FRT-CD2- FRT-Gal4/TM6*, *Hu*, *Tb* genotype were crossed to *y*^*[1]*^ *w*^[67c23];^ *snail*-cDNA (#109121 from Tokyo Stock Center) or *y*^*[1]*^ *w*^*[67c23]*^ (control) flies. The progeny of the two crosses was collected and reared on standard agar-cornmeal medium at RT until animals reached the early L1 stage, at 40 hr AED [58]. Larvae were then subjected to a heat shock at 37°C for 40 min followed by 1 hr of recovery at RT. Recovered animals were allowed to develop at 25°C for 4 d, and the non-*Tb* larvae were dissected at the end of the larval stage to examine the RGs.

## Supporting information

S1 Fig Examining *snail* expression in the PG using *snail-gfp* transgenic flies. Immunofluorescent images of RGs dissected from a transgenic line carrying the *GFP*-tagged genomic *snail* construct at various time points during the L2 stage **(A)** as well as the L3 stage **(B)**. PG and CA are outlined with a white dotted line, where CA usually lies in the middle of the tissue. Scale bar represents 50 μm. CA, corpus allatum; L2, second instar; L3, third instar; PG, prothoracic gland; RG, ring gland.
(TIF)

S2 Fig *Snail* expression levels in PG>*snail*-RNAi and RG morphology in *sna*-Enh^ΔRG^ animals. **(A)** qPCR analysis showing expression level of *snail* in the RGs. RGs were dissected at 24 hr after L2 to L3 molt. The expression in *snail*-RNAi samples were normalized to that in the control. Error bars show the 95% confidence interval. Ctrl: *UAS*-*Dicer2*; *phm22*-*Gal4*>*w*^*1118*^. *snail*-RNAi: *UAS*-*Dicer2*; *phm22*-*Gal4*>*UAS*-*snail-*RNAi. **(B)** Immunofluorescent staining of Snail in the RG. PG cells were labeled with GFP. The PG and CA are outlined by a white dotted line. *snail*-RNAi: *UAS*-*Dicer2*; *phm22*-*Gal4*>*UAS*-*snail-*RNAi; *UAS*-*EGFP*. Control: *UAS*-*Dicer2*; *phm22*-*Gal4*>*UAS*-*EGFP*. **(C)** RG morphology in *sna*-Enh^FRT^(control) and *sna*-Enh^ΔRG^ animals. Red dotted line marks the RG area. Nuclei were stained with DAPI (in gray). CA area was characterized by the membrane presence of Cadherin-N (in green). Scale bar: 10 μm. The PG area was estimated using the Z-stack images by subtracting the CA area from the RG area, and the resulted PG area was plotted in **(D)**. The ratio of PG area to CA area was plotted in **(E)**. *p < 0.05, **p < 0.01, and ****p < 0.0001. Underlying data for this figure can be found in S2 Data. CA, corpus allatum; L2, second instar; L3, third instar; PG, prothoracic gland; qPCR, quantitative PCR; RG, ring gland.
(TIF)

S3 Fig *CycE* overexpression in the PG increased the cell number of the tissue. **(A)** Maximal projection of Z-stack confocal images showing PG nuclei with DAPI staining. The PG and CA are outlined by a white dotted line. **(B)** PG cell numbers were quantified and presented in box plot. **p < 0.01. Underlying data for this figure can be found in S2 Data. CA, corpus allatum; *CycE*, *Cyclin* E; PG, prothoracic gland.
(TIF)

S4 Fig PG>*snail*-RNAi phenotype cannot be rescued by promoting S-phase entry. Bar graph shows percent survival at each indicated developmental stage in the PG>*snail*-RNAi alone or PG>*snail*-RNAi + *CycE*. Genotype tested: (1) PG>*UAS*-*snail*-RNAi; *UAS*-*EGFP*: *UAS*-*snail* RNAi/+; *UAS*-*EGFP*/*UAS*-*Dicer2*; *phm22*-*Gal4*/+. (2) PG>*UAS*-*snail*-RNAi; *UAS*-*CycE*: *UAS*-*snail*-RNAi/+; *UAS*-*Dicer2*/+; *phm22*-*Gal4*/*UAS*-*CycE*. Underlying data for this figure can be found in S2 Data. *CycE*, *Cyclin* E; L1, first instar larvae; L2, second instar larvae; L3, third instar larvae; PP, pupae.
(TIF)

S5 Fig Determining the time of CW attainment. **(A)** A schematic illustration of how to determine whether larvae have attained CW (technically minimal viable weight). The CW for metamorphosis was determined by starving L3 larvae of known developmental time classes. If starvation occurs before the larvae attained CW, development will stop and larvae do not form pupae, whereas when starvation occurs after CW checkpoint, larvae could pupariate. **(B)** Percentage of larvae that can pupariate when starvation started at various developmental time points at 25°C. The genotype tested was *UAS*-*Dicer2*; *phm22*-*Gal4*>*UAS*-*EGFP*. **(C)** Percentage of larvae that can pupariate when starvation started at various developmental stages at 18°C. The genotype tested was *UAS*-*snail*-RNAi; *tub-Gal80*^*ts*^>*phm22*-Gal4 (*snail*-RNAi was not expressed at 18°C). (B and C) The dotted line marks the cutoff for 50% pupariation. Error bar represents standard deviation. Underlying data for this figure can be found in S2 Data. End of CW: developmental time when the entire population attained CW (also minimal viable weight). CW, critical weight; L3, third instar.
(TIF)

S6 Fig Snail levels in the PG is partially controlled by IIS signaling. **(A)** Snail levels were affected by *Pten* overexpression, but not by *InR*^*DN*^ and *Akt-*RNAi. Control: *P0206*-*Gal4*>*w*^*1118*^; *UAS*:*mCD8*::*GFP*. *InR*^*DN*^: *P0206*-*Gal4*>*UAS*:*mCD8*::*GFP*; *UAS*-*InR*^*DN*^. *Pten*: *P0206*-*Gal4*>*UAS*:*mCD8*::*GFP*; *UAS*-*Pten*-cDNA. *Akt1*-RNAi: *P0206*-*Gal4*>*UAS*:*mCD8*::*GFP*; *UAS*-*Akt1*-RNAi. Five to 10 samples were examined for each condition. **(B)** Box plot showing the average of Snail nuclear fluorescent intensity examined in both control (*P0206*-*Gal4*>*w*^*1118*^; *UAS*:*mCD8*::*GFP*) and *Pten* overexpression (*P0206*-*Gal4*>*UAS*:*mCD8*::*GFP*; *UAS*-*Pten*-cDNA) PGs. *p < 0.05; (Student *t* test). **(C)** Expression of *Pi3K-* RNAi in the PG partially affected Snail levels. Eleven control samples (*phm22*>*w*^*1118*^) and 15 RNAi samples (*phm22*>*Pi3K*-RNAi) were examined. Nine out of 15 RNAi samples showed relatively normal Snail levels (upper panel), whereas six out of 15 samples showed reduced Snail levels (lower panel). (A and C) Tissues were stained with anti-Snail antibody as well as DAPI to indicate the nuclei. The PG and CA are outlined by a white dotted line. Scale bars: 50 μm for all images. Underlying data for this figure can be found in S2 Data. CA, corpus allatum; IIS, insulin/IGF signaling; *InR*^*DN*^, dominant-negative form of the insulin receptor; PG, prothoracic gland; RNAi, RNA interference.
(TIF)

S7 Fig Three proapoptotic genes are down-regulated by *snail* overexpression. qPCR analysis showing expression level of *hid*, *rpr*, and *Dcp-1* in *snail*-overexpression RGs. RGs were dissected at 24 hr after L2 to L3 molt. The expression of each gene in the *snail*-overexpression samples (*hs*-*Gal4*> *y*^*[1]*^w^*[67c23]*^, *snail-*cDNA) was normalized to the expression in the control (*hs*-*Gal4*> *y*^*[1]*^w^*[67c23]*^). Error bar show the 95% confidence interval. *p < 0.05. Underlying data for this figure can be found in S2 Data. *Dcp-1*, *Death caspase-1*; *hid*, *head involution defective*; L2, second instar; L3, third instar; qPCR, quantitative PCR; RG, ring gland; *rpr*, *reaper*.
(TIF)

S1 Table Genes that are >3-fold differentially expressed in PG>*snail*-RNAi ring glands. (XLSX)

S2 Table Top 100 differentially expressed genes in the PG>*snail*-RNAi ring glands. (XLSX)

S3 Table Top 100 differentially expressed genes in the hs>*snail*-cDNA ring glands. (XLSX)

S4 Table Genes that are >3-fold differentially expressed in hs>*snail*-cDNA ring glands. (XLSX)

S5 Table Predicted Snail binding sites for the six major ecdysone biosynthetic genes in *D. melanogaster* using INSECT 2.0. (XLSX)

S6 Table Predicted Snail binding sites for *D. melanogaster dup* using INSECT 2.0. *dup, double parked*. (XLSX)

S7 Table Primers used in the study. (XLSX)

S1 Text Supporting methods. (DOCX)

S1 Data Numerical data related to the main figures. (XLSX)

S2 Data Numerical data related to the supporting information. (XLSX)

## Abbreviations

7DC: 7-dehydrocholesterol
20E: 20-hydroxyecdysone
AED: after egg deposition
BDTNP: Berkeley *Drosophila* Transcription Network Project
CA: corpus allatum
CC: corpus cardiacum
ChIP: chromatin immunoprecipitation
C-value: DNA content value
CW: critical weight
CycE: *Cyclin* E
Dib: Disembodied
dup: *double parked*
dsRNA: double-strand RNA
EdU: 5-ethynyl-2′-deoxyuridine
EGFP: enhanced GFP
FDR: false discovery rate
FLP: Flippase
FRT: flippase recognition target
Gal80^ts^: Gal80 temperature-sensitive allele
GFP: green fluorescent protein
GO: gene ontology
hid: *head involution defective*
hs: heat shock–induced
IIS: insulin/IGF signaling
InR^DN^: dominant-negative form of the insulin receptor
INSECT 2.0: IN-silico SEarch for Co-occurring Transcription factors 2.0
L1: first instar
L2: second instar
L3: third instar
MAPK: mitogen-activated protein kinase
n.s.: not significant
NO: nitric oxide
Nvd: Neverland
PG: prothoracic gland
Phm: Phantom
PTTH: prothoracicotropic hormone
qPCR: quantitative PCR
RG: ring gland
RNAi: RNA interference
RNA-Seq: RNA sequencing
rpr: *reaper*
Sad: Shadow
Spok: Spookier
Sro: Shroud
TED: toxic extended domain
TGF-βB: transforming growth factor beta
TOR: target of rapamycin
TOR^DN^: dominant-negative form of TOR
TRiP: Transgenic RNAi Resource Project
UAS-TOR.TED: TED of TOR
VDRC: Vienna *Drosophila* Resource Center
vvl: *ventral veins lacking*
