# Supplementary figures and images for: Snail synchronizes endocycling in a TOR-dependent manner to coordinate entry and escape from endoreplication pausing during the Drosophila critical weight checkpoint
Source: PLoS Biol. 2020 Feb 25;18(2):e3000609. doi: 10.1371/journal.pbio.3000609 (PMC7041797; doi:10.1371/journal.pbio.3000609)

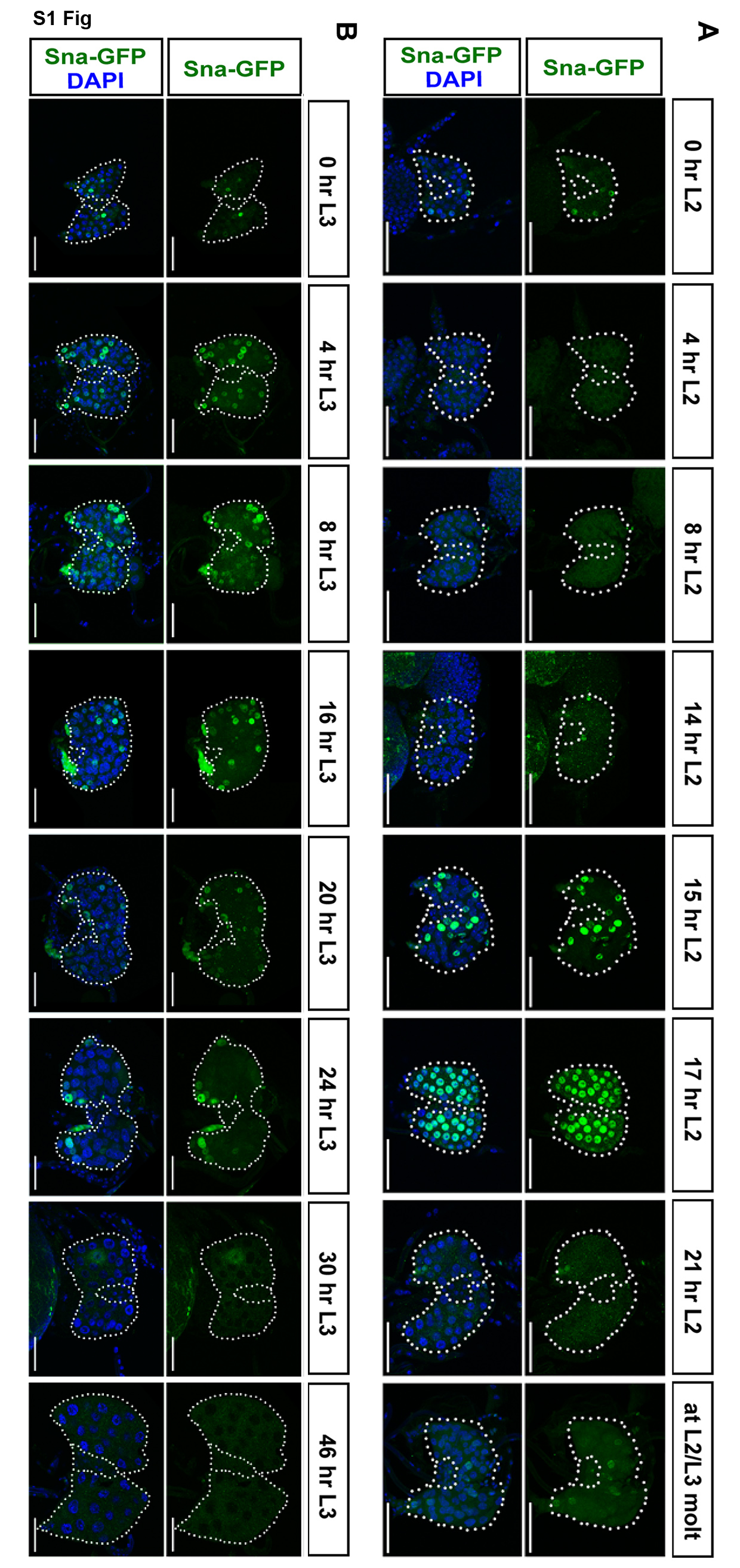

Supplement: S1 Fig — Immunofluorescent images of RGs dissected from a transgenic line carrying the GFP-tagged genomic snail construct at various time points during the L2 stage (A) as well as the L3 stage (B). PG and CA are outlined with a white dotted line, where CA usually lies in the middle of the tissue. Scale bar represents 50 μm. CA, corpus allatum; L2, second instar; L3, third instar; PG, prothoracic gland; RG, ring gland. (TIF) [file pbio.3000609.s001.tif]

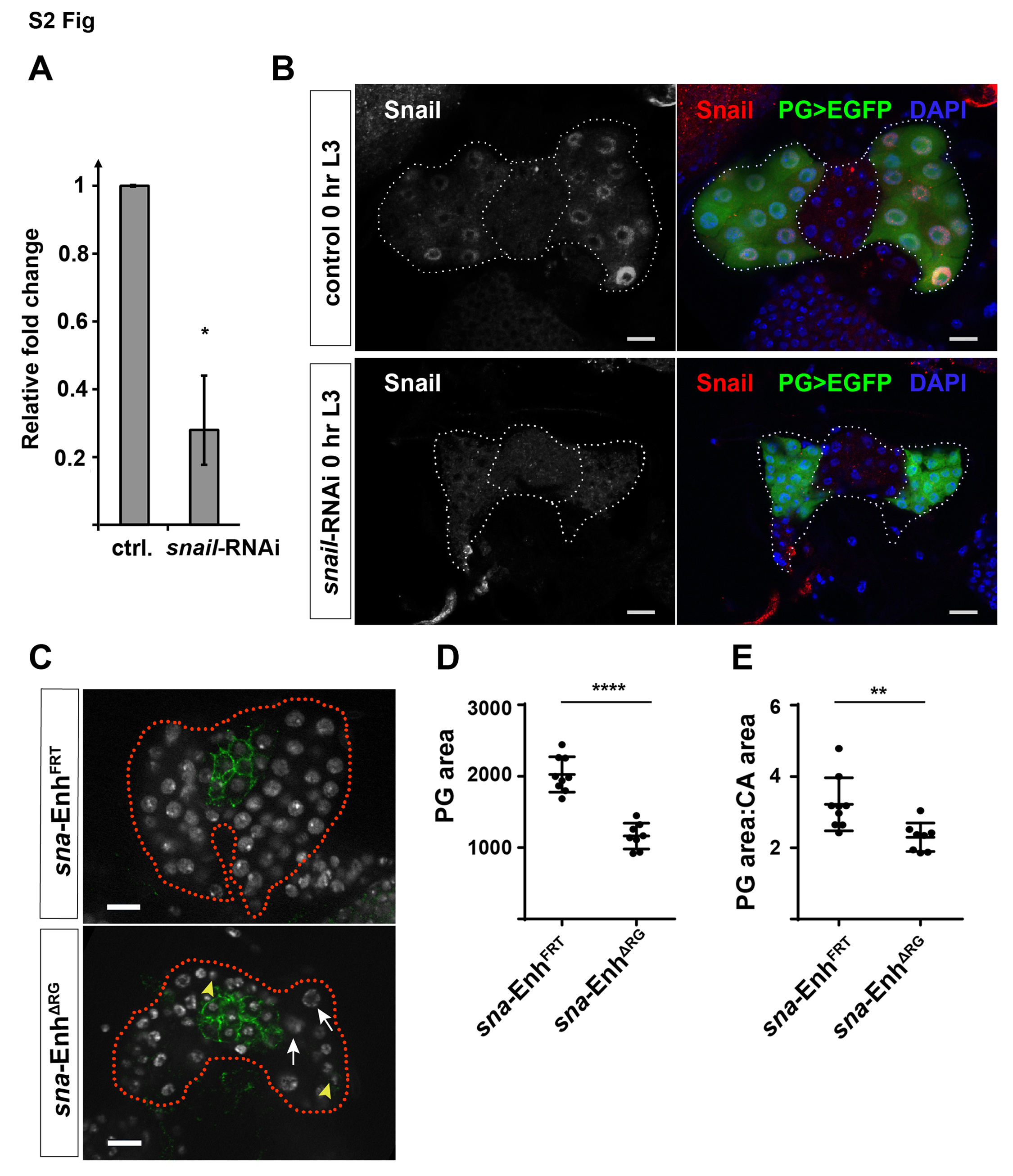

Supplement: S2 Fig — (A) qPCR analysis showing expression level of snail in the RGs. RGs were dissected at 24 hr after L2 to L3 molt. The expression in snail-RNAi samples were normalized to that in the control. Error bars show the 95% confidence interval. Ctrl: UAS-Dicer2; phm22-Gal4>w1118. snail-RNAi: UAS-Dicer2; phm22-Gal4>UAS-snail-RNAi. (B) Immunofluorescent staining of Snail in the RG. PG cells were labeled with GFP. The PG and CA are outlined by a white dotted line. snail-RNAi: UAS-Dicer2; phm22-Gal4>UAS-snail-RNAi; UAS-EGFP. Control: UAS-Dicer2; phm22-Gal4>UAS-EGFP. (C) RG morphology in sna-EnhFRT(control) and sna-EnhΔRG animals. Red dotted line marks the RG area. Nuclei were stained with DAPI (in gray). CA area was characterized by the membrane presence of Cadherin-N (in green). Scale bar: 10 μm. The PG area was estimated using the Z-stack images by subtracting the CA area from the RG area, and the resulted PG area was plotted in (D). The ratio of PG area to CA area was plotted in (E). *p < 0.05, **p < 0.01, and ****p < 0.0001. Underlying data for this figure can be found in S2 Data. CA, corpus allatum; L2, second instar; L3, third instar; PG, prothoracic gland; qPCR, quantitative PCR; RG, ring gland. (TIF) [file pbio.3000609.s002.tif]

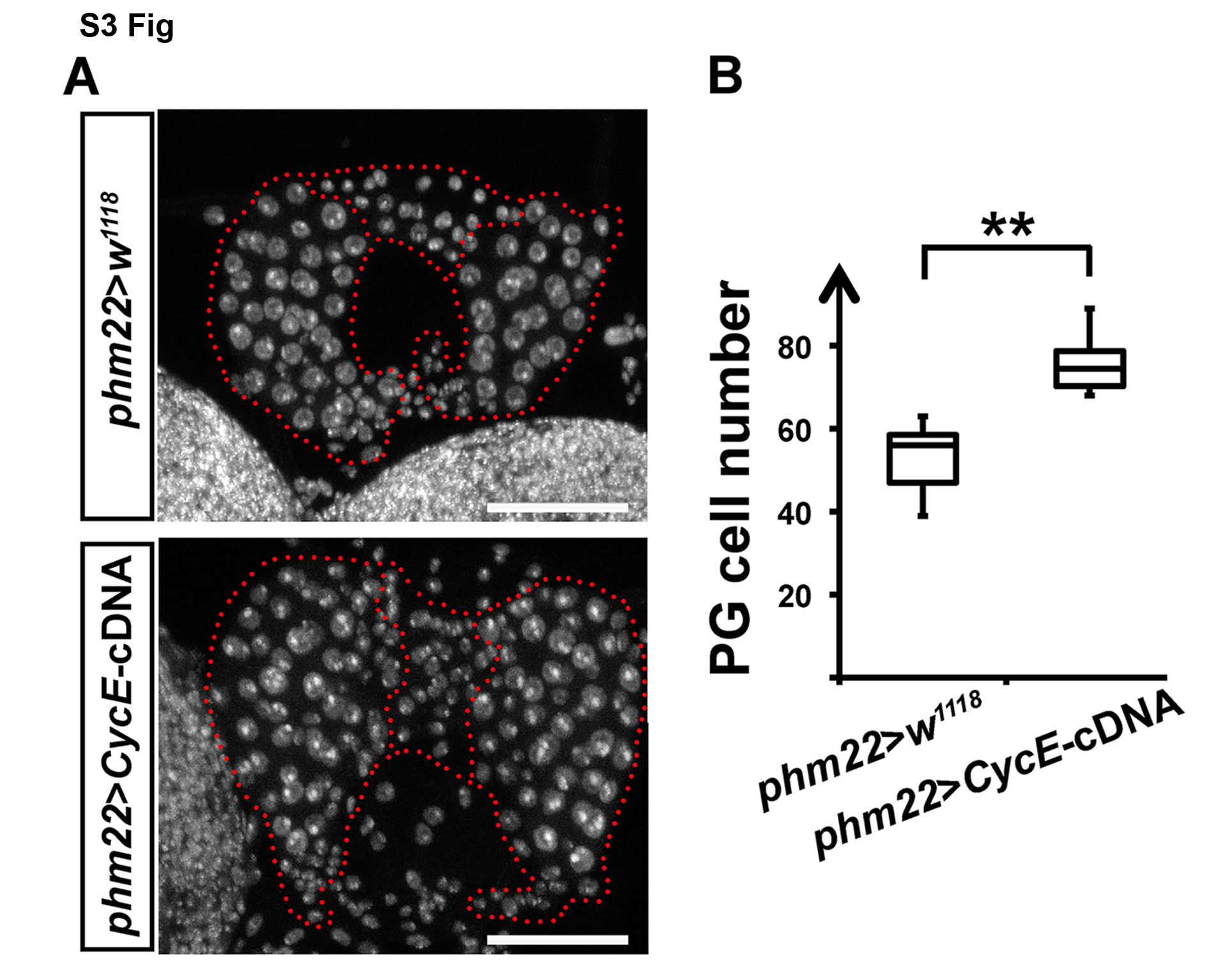

Supplement: S3 Fig — (A) Maximal projection of Z-stack confocal images showing PG nuclei with DAPI staining. The PG and CA are outlined by a white dotted line. (B) PG cell numbers were quantified and presented in box plot. **p < 0.01. Underlying data for this figure can be found in S2 Data. CA, corpus allatum; CycE, Cyclin E; PG, prothoracic gland. (TIF) [file pbio.3000609.s003.tif]

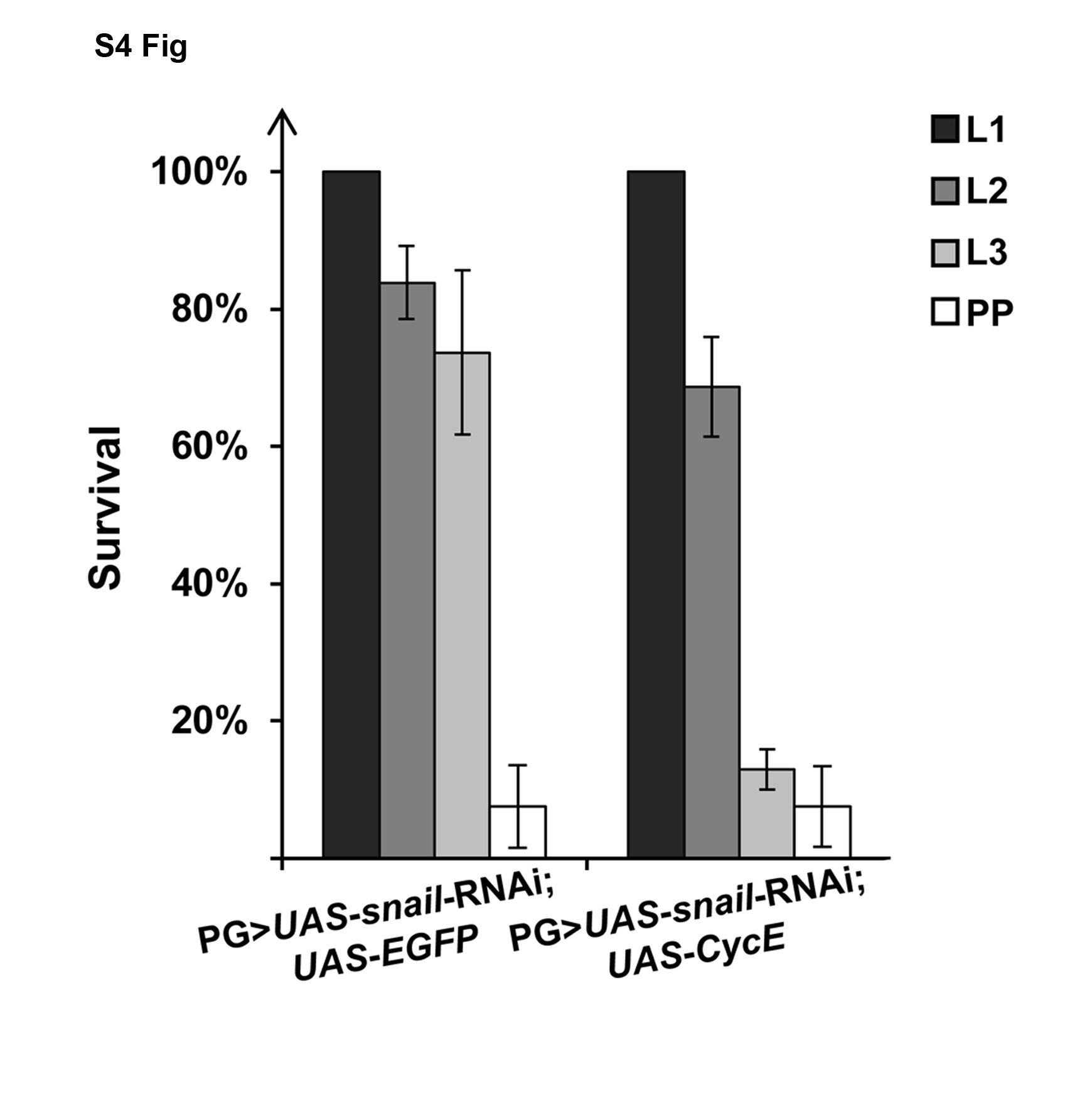

Supplement: S4 Fig — Bar graph shows percent survival at each indicated developmental stage in the PG>snail-RNAi alone or PG>snail-RNAi + CycE. Genotype tested: (1) PG>UAS-snail-RNAi; UAS-EGFP: UAS-snail RNAi/+; UAS-EGFP/UAS-Dicer2; phm22-Gal4/+. (2) PG>UAS-snail-RNAi; UAS-CycE: UAS-snail-RNAi/+; UAS-Dicer2/+; phm22-Gal4/UAS-CycE. Underlying data for this figure can be found in S2 Data. CycE, Cyclin E; L1, first instar larvae; L2, second instar larvae; L3, third instar larvae; PP, pupae. (TIF) [file pbio.3000609.s004.tif]

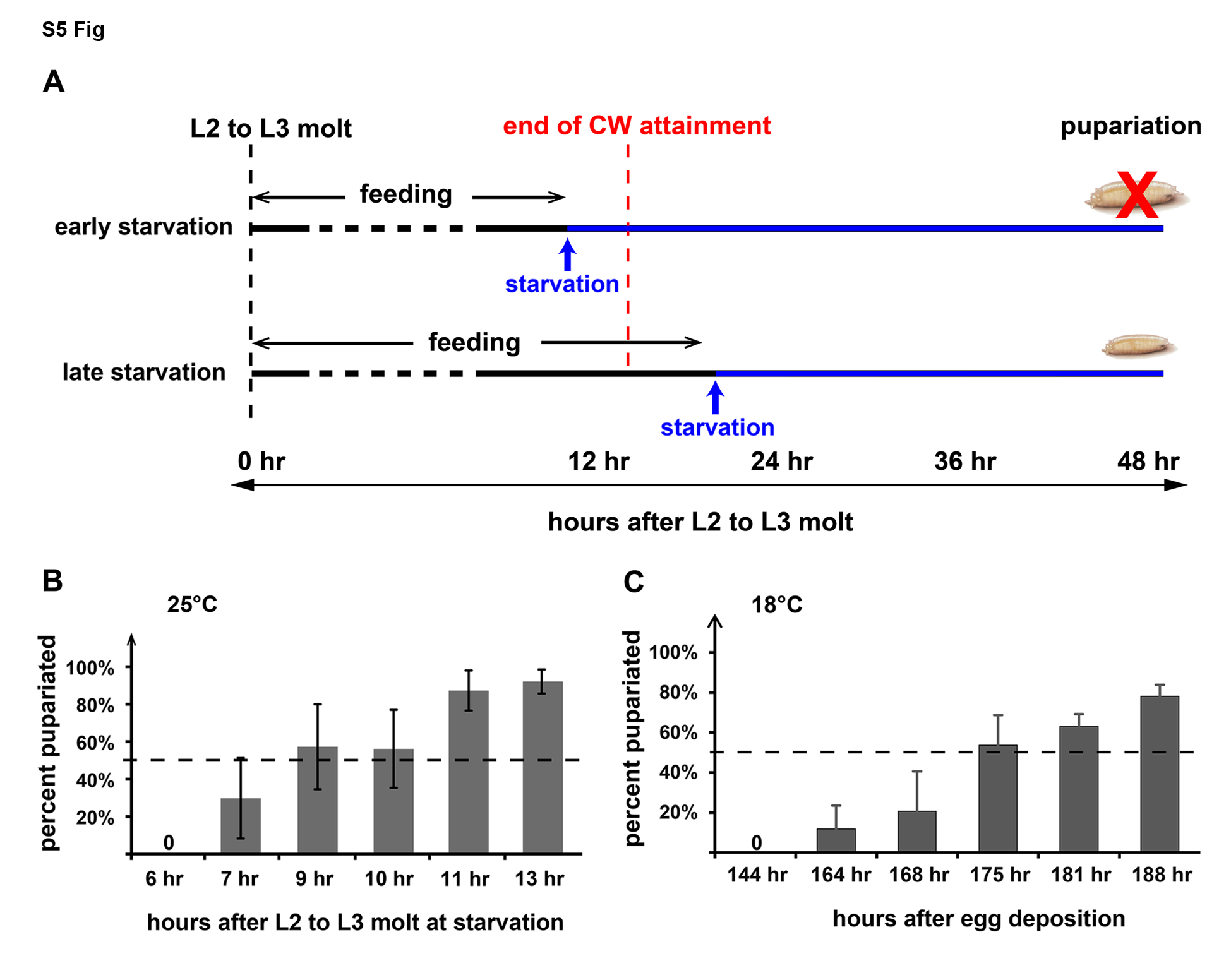

Supplement: S5 Fig — (A) A schematic illustration of how to determine whether larvae have attained CW (technically minimal viable weight). The CW for metamorphosis was determined by starving L3 larvae of known developmental time classes. If starvation occurs before the larvae attained CW, development will stop and larvae do not form pupae, whereas when starvation occurs after CW checkpoint, larvae could pupariate. (B) Percentage of larvae that can pupariate when starvation started at various developmental time points at 25°C. The genotype tested was UAS-Dicer2; phm22-Gal4>UAS-EGFP. (C) Percentage of larvae that can pupariate when starvation started at various developmental stages at 18°C. The genotype tested was UAS-snail-RNAi; tub-Gal80ts>phm22-Gal4 (snail-RNAi was not expressed at 18°C). (B and C) The dotted line marks the cutoff for 50% pupariation. Error bar represents standard deviation. Underlying data for this figure can be found in S2 Data. End of CW: developmental time when the entire population attained CW (also minimal viable weight). CW, critical weight; L3, third instar. (TIF) [file pbio.3000609.s005.tif]

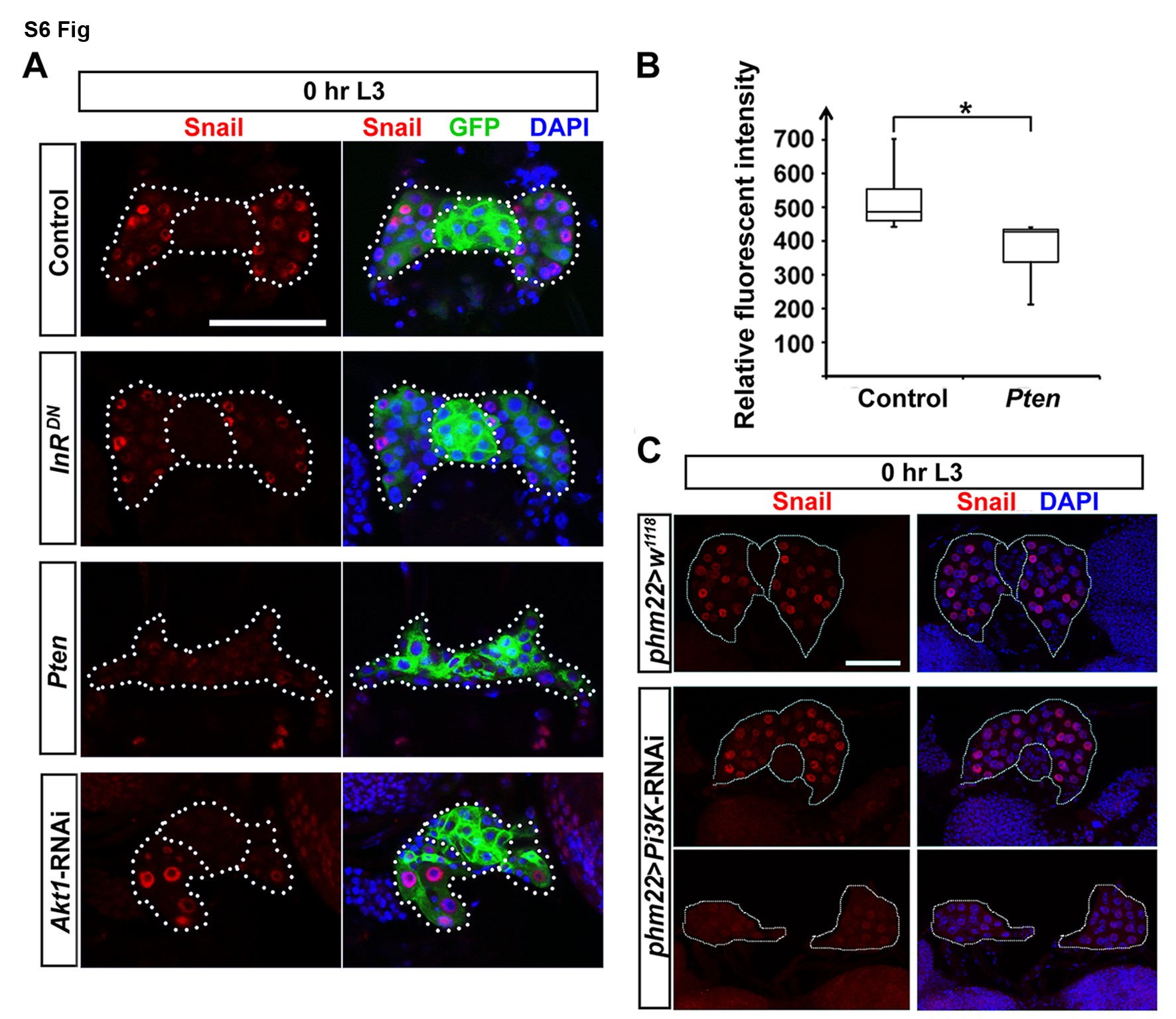

Supplement: S6 Fig — (A) Snail levels were affected by Pten overexpression, but not by InRDN and Akt-RNAi. Control: P0206-Gal4>w1118; UAS:mCD8::GFP. InRDN: P0206-Gal4>UAS:mCD8::GFP; UAS-InRDN. Pten: P0206-Gal4>UAS:mCD8::GFP; UAS-Pten-cDNA. Akt1-RNAi: P0206-Gal4>UAS:mCD8::GFP; UAS-Akt1-RNAi. Five to 10 samples were examined for each condition. (B) Box plot showing the average of Snail nuclear fluorescent intensity examined in both control (P0206-Gal4>w1118; UAS:mCD8::GFP) and Pten overexpression (P0206-Gal4>UAS:mCD8::GFP; UAS-Pten-cDNA) PGs. *p < 0.05; (Student t test). (C) Expression of Pi3K- RNAi in the PG partially affected Snail levels. Eleven control samples (phm22>w1118) and 15 RNAi samples (phm22>Pi3K-RNAi) were examined. Nine out of 15 RNAi samples showed relatively normal Snail levels (upper panel), whereas six out of 15 samples showed reduced Snail levels (lower panel). (A and C) Tissues were stained with anti-Snail antibody as well as DAPI to indicate the nuclei. The PG and CA are outlined by a white dotted line. Scale bars: 50 μm for all images. Underlying data for this figure can be found in S2 Data. CA, corpus allatum; IIS, insulin/IGF signaling; InRDN, dominant-negative form of the insulin receptor; PG, prothoracic gland; RNAi, RNA interference. (TIF) [file pbio.3000609.s006.tif]

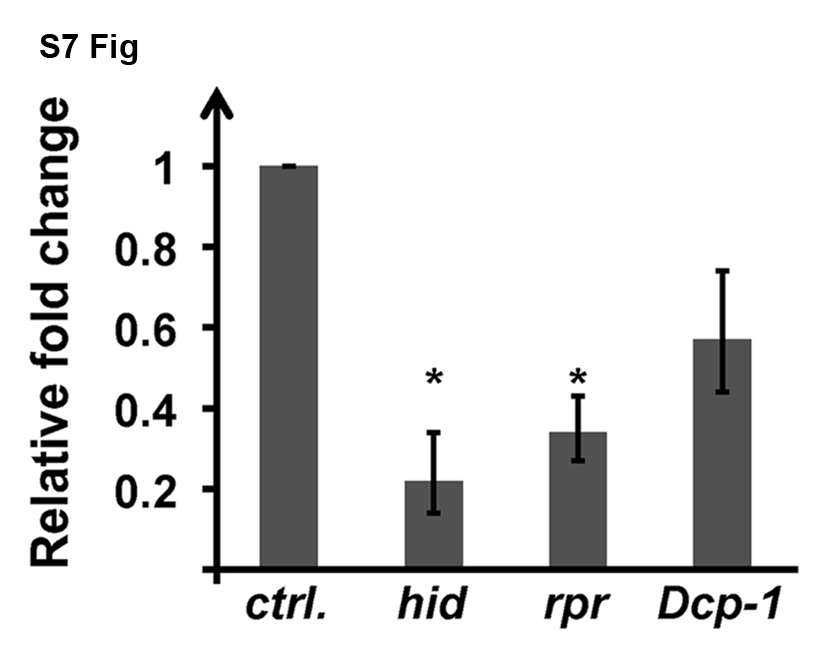

Supplement: S7 Fig — qPCR analysis showing expression level of hid, rpr, and Dcp-1 in snail-overexpression RGs. RGs were dissected at 24 hr after L2 to L3 molt. The expression of each gene in the snail-overexpression samples (hs-Gal4> y[1]w[67c23], snail-cDNA) was normalized to the expression in the control (hs-Gal4> y[1]w[67c23]). Error bar show the 95% confidence interval. *p < 0.05. Underlying data for this figure can be found in S2 Data. Dcp-1, Death caspase-1; hid, head involution defective; L2, second instar; L3, third instar; qPCR, quantitative PCR; RG, ring gland; rpr, reaper. (TIF) [file pbio.3000609.s007.tif]
