# Supplementary material for: Snail synchronizes endocycling in a TOR-dependent manner to coordinate entry and escape from endoreplication pausing during the Drosophila critical weight checkpoint
Source: PLoS Biol. 2020 Feb 25;18(2):e3000609. doi: 10.1371/journal.pbio.3000609 (PMC7041797; doi:10.1371/journal.pbio.3000609)
Supplement: S1 Text — (DOCX) [file pbio.3000609.s015.docx]

**S1 Text. Supporting Methods.**

**Heat shock induction of *snail* overexpression**

hs-Gal4 (Bloomington #2077) was crossed with *y[1] w[67c23]*; *snail*-cDNA lines and were kept at RT until induction. The progeny was heat-shocked at 17 hr after the L2 to L3 molt for 45 min in a 37°C water bath and allowed to recover for 6 hours at 25°C before RNA extraction for RNA-Seq experiments. hs-Gal4> *y[1] w[67c23]*, which served as controls were treated in the same manner.

**Cloning of donor template for CRISPR-Cas9 meditated HDR**

A ~1.25-kb sequence upstream the first *snail* enhancer gRNA cleavage site (fragment 1, S7 Table) was PCR amplified (Phusion, New England BioLabs) and then cloned into the pHD-dsRed-attp vector between EcoRI and NdeI using conventional cloning. And this fragment 1 will serve as the left homology arm. The first FRT site was integrated into fragment 1 through the reverse primer. Fragment 2 contains the region between -6421bp to -3443bp relative to *snail* TSS where the second FRT site was included at the end (3’) of the fragment. Another ~1.13kb sequence downstream the second FRT insertion site in the genome was amplified as the right homology arm (fragment 3). Fragment 2, fragment 3 and the linearized vector (via SpeI and XhoI digestion) were assembled using Gibson Assembly (New England BioLabs). All primers used for cloning are listed in S7 Table.

**Cellular DNA content measurements**

Cellular DNA content was assessed by DAPI staining (1:50,000). Z-stack images of the brain-RGs complexes were taken, and the summation of DAPI intensity for every single pixel of all the stacks was obtained using ImageJ. Corrected DAPI intensity (CDI) in the PG area was calculated using the following formula: CDI = Summation of DAPI intensity of the selected area – (Area * Mean intensity of background readings). All the parameters in the formula were measured in ImageJ. Next, the CDI for the PG area was normalized to the average DAPI intensity in the brain lobe, and the normalized DAPI intensity was divided by PG cell number to obtain relative DNA intensity per cell.

**Starvation protocol for critical weight determination**

Whether a larva had passed the CW checkpoint was estimated by whether the larva could pupariate when deprived of food (technically MVW). Briefly, larvae were staged at the L2/L3 molt. A population of larvae with the same developmental age was transferred to starvation medium with only 2% agar in water, and the fraction of pupariation was scored in the following two days.
